# Strand-Specific RNA-Seq Analyses of Fruiting Body Development in *Coprinopsis cinerea*

**DOI:** 10.1371/journal.pone.0141586

**Published:** 2015-10-28

**Authors:** Hajime Muraguchi, Kiwamu Umezawa, Mai Niikura, Makoto Yoshida, Toshinori Kozaki, Kazuo Ishii, Kiyota Sakai, Motoyuki Shimizu, Kiyoshi Nakahori, Yuichi Sakamoto, Cindy Choi, Chew Yee Ngan, Eika Lindquist, Anna Lipzen, Andrew Tritt, Sajeet Haridas, Kerrie Barry, Igor V. Grigoriev, Patricia J. Pukkila

**Affiliations:** 1 Department of Biotechnology, Faculty of Bioresource Sciences, Akita Prefectural University, Akita, 010-0195, Japan; 2 Department of Environmental and Natural Resource Science, Faculty of Agriculture, Tokyo University of Agriculture and Technology, Fuchu, Tokyo, 183-8509, Japan; 3 Department of Applied Biological Science, Faculty of Agriculture, Tokyo University of Agriculture and Technology, Fuchu, Tokyo, 183-8509, Japan; 4 Department of Applied Biological Chemistry, Faculty of Agriculture, Meijo University, Nagoya, Aichi, 468-0073, Japan; 5 Graduate School of Natural Science and Technology, Okayama University, Okayama, 700-8530, Japan; 6 Iwate Biotechnology Research Center, Kitakami, Iwate, 024-0003, Japan; 7 US Department of Energy Joint Genome Institute, 2800 Mitchell Drive, Walnut Creek, CA, 94598, United States of America; 8 Department of Biology, University of North Carolina at Chapel Hill, Chapel Hill, NC, 27599-3280, United States of America; Friedrich Schiller University, GERMANY

## Abstract

The basidiomycete fungus *Coprinopsis cinerea* is an important model system for multicellular development. Fruiting bodies of *C*. *cinerea* are typical mushrooms, which can be produced synchronously on defined media in the laboratory. To investigate the transcriptome in detail during fruiting body development, high-throughput sequencing (RNA-seq) was performed using cDNA libraries strand-specifically constructed from 13 points (stages/tissues) with two biological replicates. The reads were aligned to 14,245 predicted transcripts, and counted for forward and reverse transcripts. Differentially expressed genes (DEGs) between two adjacent points and between vegetative mycelium and each point were detected by Tag Count Comparison (TCC). To validate RNA-seq data, expression levels of selected genes were compared using RPKM values in RNA-seq data and qRT-PCR data, and DEGs detected in microarray data were examined in MA plots of RNA-seq data by TCC. We discuss events deduced from GO analysis of DEGs. In addition, we uncovered both transcription factor candidates and antisense transcripts that are likely to be involved in developmental regulation for fruiting.

## Introduction

**Fig 1:**
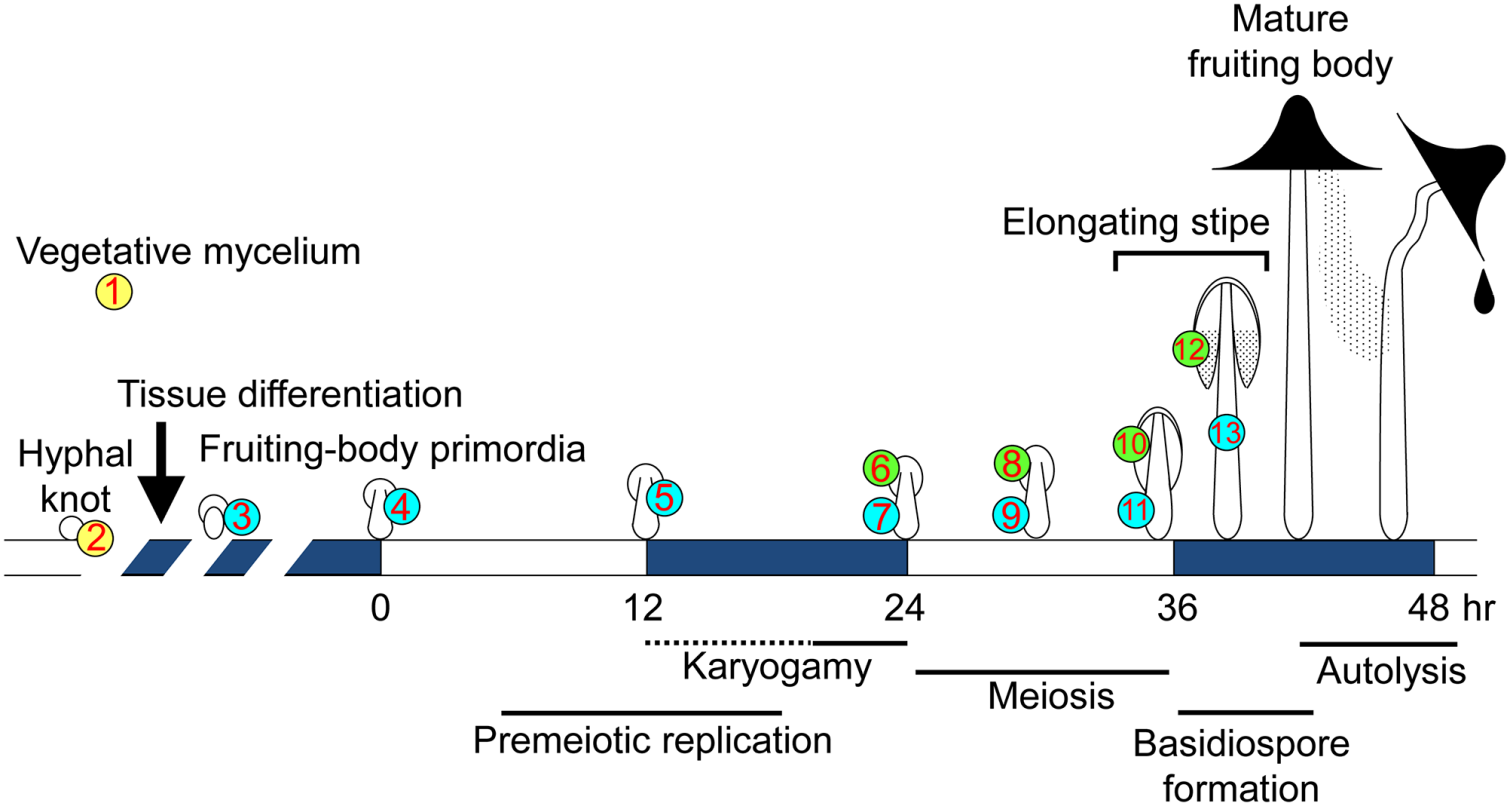
Schematic diagram of fruiting body development in *C*. *cinerea*. The 13 stages/tissues (numbers in circles) were selected to investigate the transcriptome by RNA-seq. Developmental and cellular events in each stage/tissue are shown in Table 1.

The basidiomycete fungus *Coprinopsis cinerea* produces highly differentiated multicellular structures, fruiting bodies, providing an important model system for multicellular development [1, 2]. The 13 stages/tissues sampled for this work are depicted in Fig 1 and the developmental changes during each stage are summarized in Table 1. Fruiting body formation begins with an aggregation of hyphae, leading to hyphal knots of about 0.2 mm or less in diameter. At the core of the hyphal knots, hyphal growth with nuclear divisions rapidly occurs, resulting in highly branched short cells and an increase in cell density [3, 4]. The surface of the hyphal knots becomes covered by a layer of veil cells. One side of the hyphal knot differentiates into the primordial shaft, followed by differentiation of the rudimentary pileus (cap) at the opposite side of the primordial shaft, forming a tiny fruiting body primordium [1, 3, 4]. The gills develop on the underside of the pileus, and basidial cells differentiate on the surface of the gills [5]. The primordium gradually enlarges and matures under proper light conditions, such as the 12 hr light/ 12 hr dark cycle [6, 7]. The maturation stage is triggered by light (0 hr in Fig 1), in which premeiotic DNA replication starts in basidial cells, followed by karyogamy (the fusion of compatible haploid nuclei) [8]. After karyogamy, meiosis proceeds in the following light period [6, 9, 10]. The stipe cells, in which nuclei divide without cell division [11, 12], start to elongate around the end of meiosis [13]. As the stipe elongates, the basidiospores are produced on the outside of each basidium, arrayed on the surface of gills, making the underside of the pileus black, due to color of the mature basidiospores.

**Table 1:** Samples used for RNA-seq.

Point#	Abbreviation	Stage/Tissue	Developmental and cellular events
1	(My)	Vegetative mycelium	Tip growth, Aging
2	(Knot)	Hyphal knots with vegetative mycelium	Differentiation of core and veil cells
3	(sPri)	Small fruiting body primordia	Differentiation of primordial shaft and primitive hymenium
4	(0hrPri)	Fruiting body primordia at 0hr*	Light triggers the maturation stage.
5	(12hrPri)	Fruiting body primordia at 12hr after the trigger light	Light is received for 12 hr. Premeiotic DNA replication starts.
6	(24hrCap)	Cap at 24hr after the trigger light	Karyogamy (K) occurs in basidial cells.
7	(24hrStipe)	Stipe at 24hr after the trigger light	Stipe before enlargement. Nucleus divides in stipe cells.
8	(30hrCap)	Cap at 30hr after the trigger light	K+6 stage
9	(30hrStipe)	Stipe at 30hr after the trigger light	Stipe enlarges.
10	(36hrCap)	Cap at 36hr after the trigger light	K+12 stage
11	(36hrStipe)	Stipe at 36hr after the trigger light	Stipe starts to elongate.
12	(39hrCap)	Cap at 39hr after the trigger light	K+15 stage, sporogenesis occurs.
13	(39hrStipe)	Stipe at 39hr after the trigger light	Stipe is elongating. Cap is expanding.

*: 0hr means the time when light triggers the maturation stage [6].

Strain #326 is a homokaryotic strain that displays clamp formation and fruiting without mating, because this strain has mutations in both mating type factors, *A* and *B*, activating mating pathways [14]. The mutant *A43* locus in #326 has a deletion that results in a gene fusion, which is predicted to encode a chimeric HD2:HD1 protein that constitutively promotes *A*-regulated clamp cell development and fruiting [15]. The *B* mating type locus contains multi-allelic genes encoding pheromones and G-protein-coupled receptors [16]. A single amino-acid substitution in either pheromone or receptor have been reported to deregulate the specificity of ligand-receptor recognition and confer a self-compatible *B* phenotype [17]. Strain #326 has been used for isolation of mutations [18, 19], construction of a linkage map [20] and comparative transcriptomics and proteomics [21]. Some traits of #326 are somewhat different from those of the wild-type dikaryon. Unlike dikaryons, asexual spores, oidia, are produced on the vegetative mycelium of #326 [22]. However, most of the fruiting processes are common to the dikaryotic fruiting.

The genome of a homokaryotic strain, Okayama-7, was sequenced and assembled [23] and has allowed post-genome studies, including microarray analysis [24], SAGE [25], transcriptomics, proteomics [21] and epigenetics [26]. In this study, to investigate the transcriptome in detail during fruiting body development, we have sequenced the genome of strain #326, and prepared samples of total RNA with two biological replicates from 13 stages/tissues in the homokaryotic fruiting of #326. Strand-specific RNA-seq libraries were constructed, and the reads were counted against forward and reverse strands for all gene models, and mapped to the genomic sequence to observe transcripts. We report a comprehensive view of the transcriptome during fruiting body development, and focus on transcription factor candidate genes and possible antisense transcripts.

## Materials and Methods

### Strain and culture conditions

The homokaryotic fruiting strain #326 (*A43mut B43mut pab1-1*) was grown on yeast extract-malt extract-glucose (YMG) medium [27] solidified with 1.5% (w/v) agar at 28°C in a 12hr light/12hr dark regime. To obtain vegetative mycelium, an agar cube with mycelium was inoculated on the center of a cellophane sheet placed on the YMG agar plate medium, and incubated for 4 days. The mycelium was harvested by scraping gently from the cellophane sheet.

**Fig 2:**
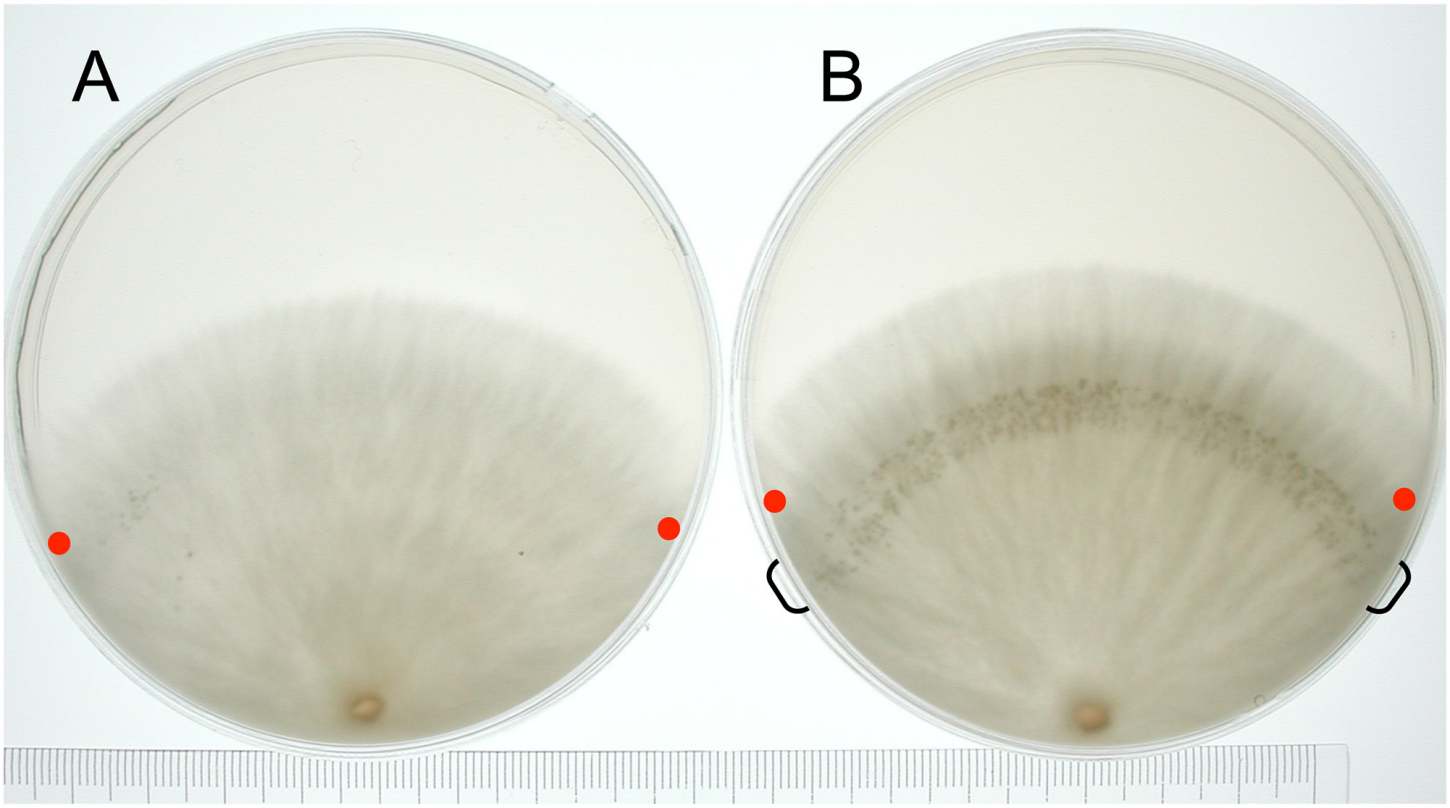
Hyphal knot induction. (A) The mycelium was cultured in the 12 hr light/ 12 hr dark cycle. (B) The mycelium was cultured in dark for 5 days, followed by 2 hr light and 24 hr dark. The hyphal knots were induced at the area indicated by parenthesis. Red dots indicate the position of apical hyphae when light was received.

To harvest hyphal knots with the vegetative mycelium, an agar cube with mycelium was also inoculated on the periphery of a cellophane sheet placed on the YM1/2G agar plate medium, which contains 0.2% glucose, instead of 0.4% glucose in YMG medium [28]. This reduction of glucose mimics the typical situation in which the glucose concentration falls as the mycelium grows on YMG medium, causing the mycelium to be highly sensitive to fruiting induction. The plates were incubated for five days in dark, for 2 hr in light, and for 24 hr in dark. After the last dark period, hyphal knots were formed in a restricted portion of the mycelium (the region that included apical hyphae during the 2 hr illumination period). As it is hard to separate hyphal knots from the vegetative mycelium, hyphal knots were harvested with the vegetative mycelium by excising the hyphal knot region (Fig 2) with a knife and scraping gently from the cellophane sheet. The tissues from 13 points shown in Fig 1 were harvested with two biological replicates and weighed. Approximately 0.2 g of the tissues were flash-frozen in liquid nitrogen and stored at –80°C for later use.

### cDNA sequencing and counting

Total RNA was extracted from each sample with an RNeasy Plant Mini kit (Qiagen), in which the first reagent of the kit (Buffer RLC) was added to the frozen tissue, and ground using a mortar and pestle. For each stage/tissue, 50 μg of total RNA were sent to the Joint Genome Institute (JGI). At JGI, mRNA was purified from total RNA using Absolutely mRNA™ purification kit (Stratagene) and chemically fragmented to 200-250bp (Ambion). mRNA was reverse transcribed with SuperScript II using random hexamers. Second strand cDNA was synthesized using dNTP/dUTP mix (Thermo Scientific), *E*. *coli* DNA Ligase, *E*. *coli* DNA polymerase I, and *E coli* RnaseH (Invitrogen). The fragments were treated with end-repair, A- tailing, and ligation of adaptors using the Illumina Truseq DNA Sample Prep Kit (Illumina). Second strand cDNA was removed by AmpErase UNG (Applied Biosystems) to generate strandedness similar to the method described by Parkhomchuk *et al*. [29] and enriched with 10 cycles of PCR to generate the final library. qPCR was used to determine the concentration of the libraries. Libraries were sequenced on the Illumina Hiseq, producing paired end reads R1 and R2 from each sample (fastq) with 100 bp in each read.

### Alignment, read counting, and DEG detection

The #326 *Amut Bmut pab1-1* genomic DNA was sequenced, assembled and used to predict 14,245 gene models (NCBI Accession PRJNA258994; S1 Text). Reads were deposited to SRA under the following accessions: SRA051294, SRA051421, and SRA050788. The paired end reads R1 and R2 from each sample (fastq) were independently aligned to the gene models using Bowtie2 [30], and counted for plus strand and minus strand, respectively. The counts for a sense transcript (Forward: Fw) were obtained as sum of R1_minus counts and R2_plus counts. The counts for an anti-transcript (Reverse: Rv) were obtained by sum of R1_plus counts and R2_minus counts. The raw count data were normalized to obtain RPKM (reads per kilobase of gene model per million mapped reads; [31]). Expression levels of sense transcripts (Fw) were given in an average of four values: two R1_minus and two R2_plus counts from two replicate samples, with standard deviation. Those of antisense transcripts (Rv) were also given in an average of four values: two R1_plus and two R2_minus counts from two replicate samples (S1 Table). The raw count data were normalized by the Tag Count Comparison (TCC) R package [32] to detect differentially expressed genes (DEG) between two stages.

To visualize antisense transcripts, the reads R1 and R2 from each sample (fastq) were independently aligned using an available gff (Copci_AmutBmut1_GeneCatalog_genes_20130522.gff) to the reference genomic sequence, (Copci_AmutBmut1_AssemblyScaffolds_Repeatmasked.fasta) by tophat-2.0.14.OSX_x86_64 [33], and the reads were separated based on read strands in the Integrative Genomics Viewer (IGV) [34].

### Microarray data analysis

Microarray data, GSE37943_RAW and GSE37942_RAW, were downloaded from GEO DataSets. Data from 44 microarrays were normalized by Variance Stabilization and Normalization (vsn) R package [35]. Based on the density plots of the normalized data, the 10 microarrays showing abnormal distributions were removed, and data from the remaining 34 microarrays were normalized again by vsn. Signals from the wild-type channels were collected in each stage and back-ground corrected by subtracting an intensity with a minimum density between two peaks in the density plot, in which each peak corresponds to empty and oligo spots, respectively. The corrected intensities were used to calculate differences between K and K+6, and K+6 and K+12. The differences were assessed by t-test.

### qRT-PCR validation

RNA-seq results were validated by quantitative real-time PCR (qRT-PCR). cDNAs for qRT-PCR were synthesized from total RNA used for RNA-seq with RevaTra Ace qPCR RT Kit (TOYOBO). The quantitative measurement of gene expression was performed with a CFX96 (Bio-Rad). The primers for qRT-PCR are listed in S2 Table. The β-tubulin gene was used as an internal control. Log_2_ ratios of expression data in both platforms were calculated and examined for correlation.

### GO analysis

Functional annotation clustering of DEGs in each transition was performed through the web-based interface of the DAVID Knowledgebase [36]. When there were more than 3000 DEGs in a transition, the top 3000 most significant genes were used.

## Results and Discussion

### 1. Overview of fruiting body development with rationale for the stages chosen

To investigate the transcriptome in detail during fruiting body development, 13 stages/tissues were chosen for sampling (Fig 1): vegetative mycelium (1_My), hyphal knots (2_Knot), small primordia (3_sPri), primordia that receive a trigger light (TL) to start the maturation stage (4_0hrPri), primordia 12 hr after TL (5_12hrPri), the cap of primordia 24 hr after TL (6_24hrCap), the stipe of primordia 24 hr after TL (7_24hrStipe), the cap of primordia 30 hr after TL (8_30hrCap), the stipe of primordia 30hr after TL (9_30hrStipe), the cap of primordia 36 hr after TL (10_36hrCap), the stipe of primordia 36 hr after TL (11_36hrStipe), the cap of primordia 39 hr after TL (12_39hrCap), the stipe of primordia 39 hr after TL (13_39hrStipe). In these stages/tissues, various cellular events occur (Table 1). The developmental lineages among the stages/tissues are shown in S1 Fig.

To harvest hyphal knots, we developed a method in which light conditions induce hyphal knots on a certain region of the vegetative mycelium (Fig 2). The mycelium was cultured in dark for five days, followed by 2 hr light and 24 hr dark. The hyphal knots were synchronously induced in the region of the mycelium where light was received.

### 2. Assessment of samples

Total RNA was extracted from each stage/tissue with two biological replicates, which were distinguished by the addition of a or b to the sample number, for example, 1a and 1b. Twenty-six cDNA libraries were constructed using strand-specific methods [29] and sequenced to produce a total 93.4 Gb of transcript reads (S1 Table). The R1 and R2 reads were mapped to total 14,245 gene models, and mapped read counts varied from 64.8% to 84.1%, and from 65.9% to 87.5%, respectively. Further analysis will be required for unmapped reads, which might include reads derived from splicing variants or isoforms.

**Fig 3:**
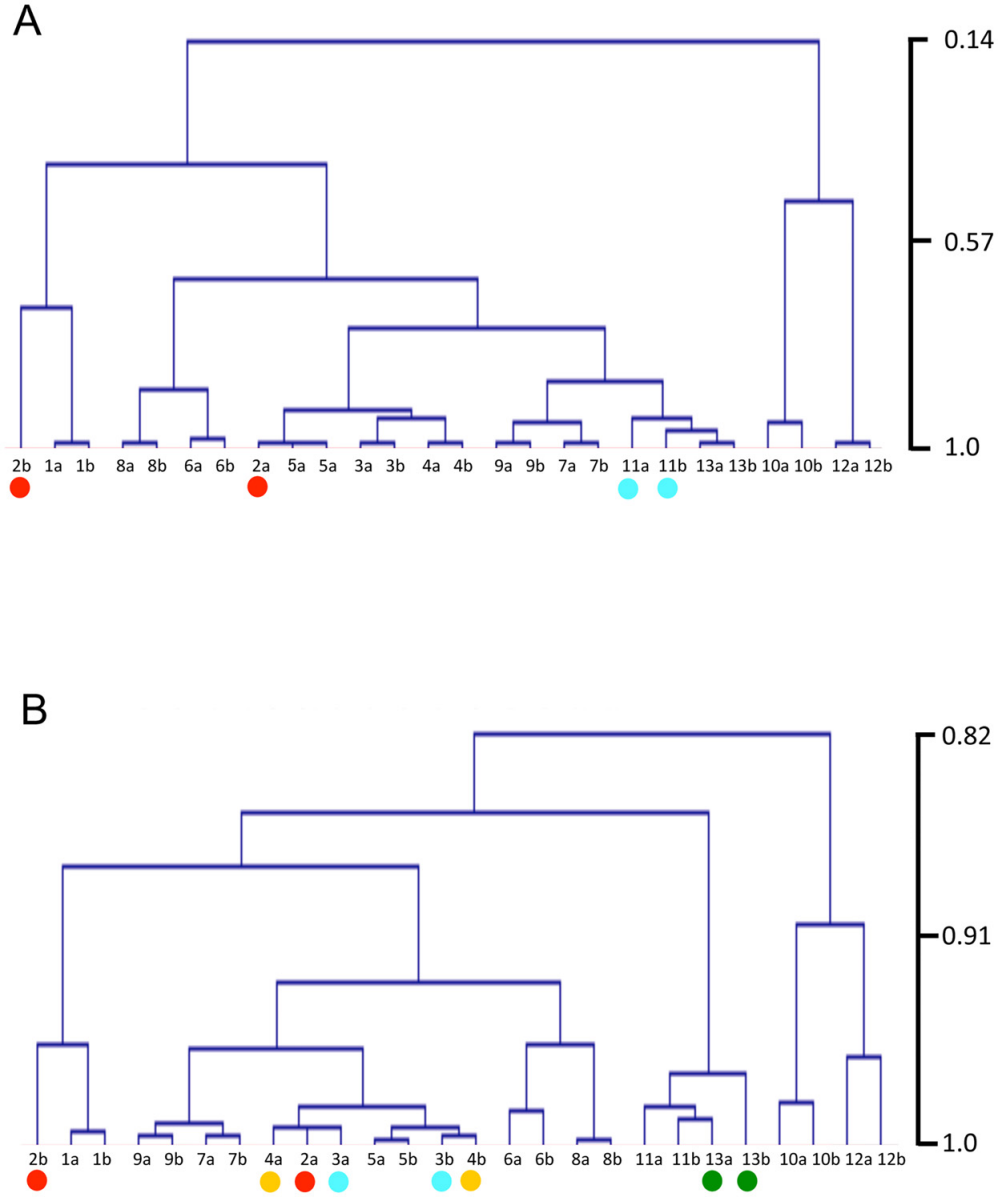
Clustering of samples using RPKM values of sense transcripts. Hierarchical clustering of samples was performed using RPKM values of sense transcripts from all gene models. The trees were depicted with MeV [63] by using average linkage distance measurement and Pearson’s correlation (A) and Spearman’s correlation (B). The vertical scale is correlation coefficient. Red dots indicate separated 2_Knot samples. Green, blue and yellow dots also indicate duplicate samples that are not located in a single clade.

To assess samples used for RNA-seq analysis, the samples were clustered using the RPKM values of all gene models in each sample with two types of correlation methods: Pearson and Spearman, and using clustering: average for each (Fig 3). Most of the replicate samples showed high correlation, but 2a_Knot and 2b_Knot were located in different clades in the trees. Since these samples contain both knots and also the vegetative mycelium, it is reasonable that the 2b_Knot stage clusters with the vegetative mycelium. In the hyphal knots, cell division and differentiation occur rapidly. This rapid transition might make it difficult to harvest the same stage in hyphal knots, resulting in the 2a_Knot sample clustering with later stages.

The clustering trees indicate that gene expression in the 10_36hrCap and 12_39hrCap samples is quite different from expression in other stages. During this period, sporogenesis occurs (Table 1), in which many metabolic changes might be required.

### 3. Detection of differentially expressed genes (DEGs)

**Fig 4:**
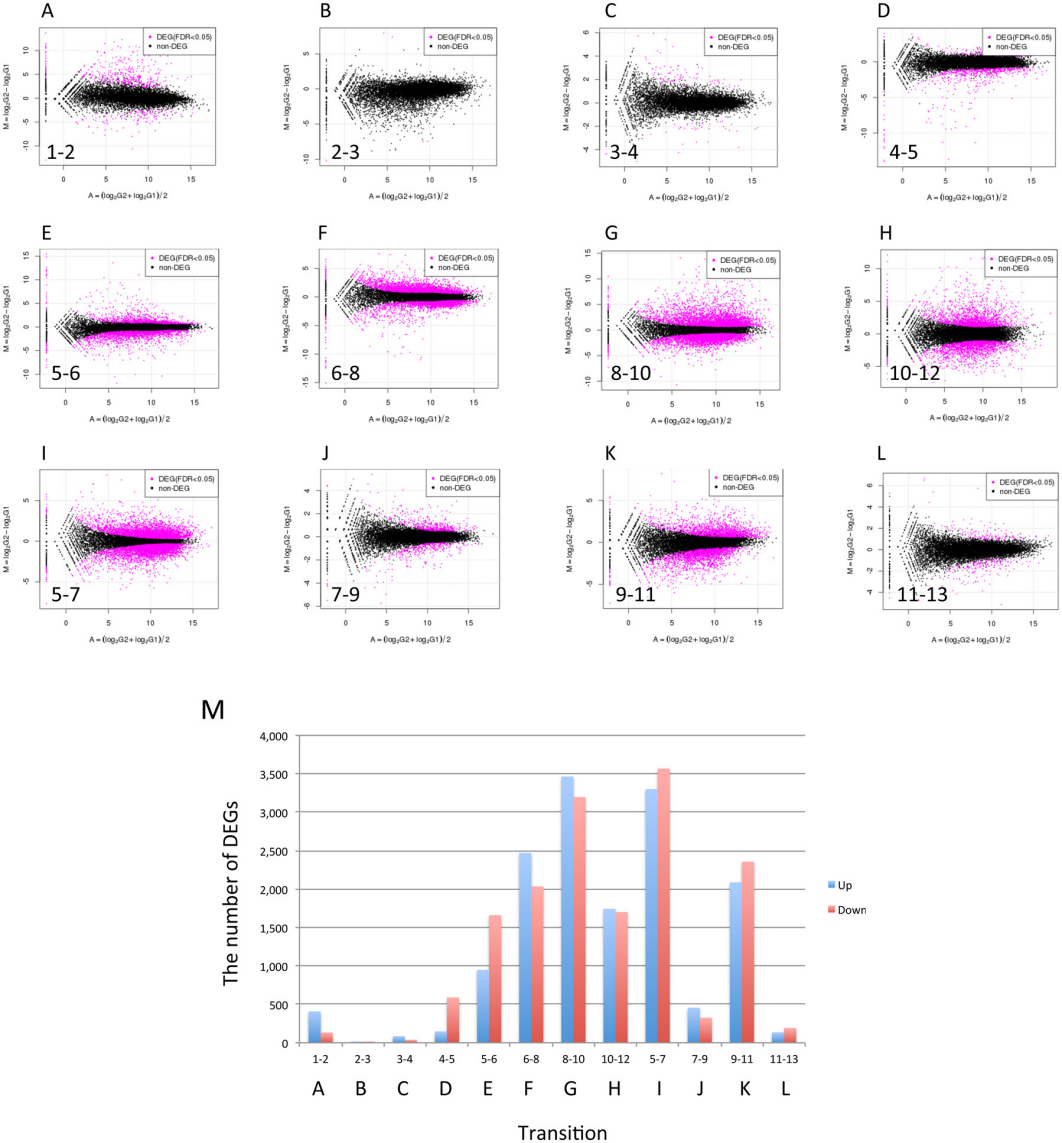
MA plots and change in DEGs for adjacent stages/tissues. (A-L) Forward counts of adjacent stages/tissues were normalized by TCC, which detected DEGs (indicated using red points). (M) Change in the number of up- and down-regulated DEGs during fruiting. Transitions, A-L, correspond to graphs of panel A-L.

Tag Count Comparison (TCC, [32]) was performed using count data of RNA-seq between adjacent stages to detect differentially expressed genes (DEGs). MA plots are shown in Fig 4A–4L, in which red dots indicate DEGs detected with a FDR <0.05. The number of up- and down-regulated DEGs in each transition is shown in Fig 4M. The largest number of up-regulated DEGs, 3,465 genes, was detected in transition from 8_30hrCap to 10_36hrCap (24.32% of 14,245 gene models, Fig 4G). The largest number of down-regulated DEGs was detected in transition from 5_12hrPri to 7_24hrStipe (25.05%, Fig 4I). In the edible mushroom *Volvariella volvacea*, the highest number of DEGs was observed in a transition between corresponding stages, egg to elongation, in which 76% of DEGs were down-regulated. In these transitions, down-regulation of gene expression would be required to promote fruiting body development. The small number of DEGs detected in transition from 2_Knot to 3_sPri is likely due to variation between replicate samples in the knot stage (Fig 3). Indeed, there are many genes that show high m.values but could not be detected as DEGs (Fig 4B).

TCC was also performed using gene expression in vegetative mycelium as a reference to detect DEGs. To show changes in gene expression, MM plots were depicted and revealed a group of genes that show characteristic changes in gene expression in a certain transition (S2 Fig). In the MM plots, the x-axis indicates m.values between 1_My and the previous stage of a certain stage (defined as the historical difference), and the y-axis indicates m.values between the certain stage and the next stage (defined as the transitional difference). One category (DEG-type A) contains genes that show no historical difference, (x = around 0), and are up-regulated in a certain transition (y > = 4). This category was observed in the 5–6, 8–10 and 10–12 transitions (S2 Fig). Another category (DEG-type B) contains genes that have high expression levels in historical difference and are down-regulated in a certain transition (x > = 4, y = < -4). This category was observed in the 4–5, 5–6 and 6–8 transitions. The top 20 DEGs estimated to have the most significant changes are listed in S3 Table. Genes in both the DEG-type A and DEG-type B categories shown in S2 Fig are identified in S3 Table.

### 4. Validation of RNA-seq data by comparison with qPCR and microarray data

To validate RNA-seq data, ratios of expression levels of selected genes (S2 Table) to that of the β-tubulin gene in 26 samples (13 stages/tissues with two biological replicates) were calculated using RPKM values of RNA-seq data and qRT-PCR data. Scatter plots of log_2_ transformed ratios were depicted (S3 Fig), in which y-intercepts in the approximation formula for each gene were corrected to 0. The ratios were well correlated in genes showing expression levels similar to the β-tubulin gene. The genes with low correlation might be present in too low a concentration for detection of expression differences in qRT-PCR.

To further validate and characterize RNA-seq data, we also compared it with microarray data published previously [24]. The microarray data were obtained using cDNA synthesized from the gill tissue to investigate the meiotic process from K (Karyogamy) to K+12 hr [24]. The gill tissue at the karyogamy stage (K) is included in 6_24hrCap (S1 Fig). Microarray data and RNA-seq data were normalized by vsn and TCC, respectively. The scatter plots were depicted using log transformed values in both platforms (S4 Fig).

In two transitions, from 6_24hrCap (K) to 8_30hrCap (K+6), and from 8_30hrCap (K+6) to 10_36hrCap (K+12), genes with 2-fold changes (m.value>1 or <-1) and significant differences in expression (p<0.05) as determined by the t-test were selected as DEGs in microarray data (S4 Table). The 45 genes in the DEGs are up-regulated between K and K+6. Among them, 42 genes (93.3%) showed similar changes to those in RNA-seq data, and two genes (4.4%) are inconsistent with changes in RNA-seq data (type A in S5 Table). Expression changes of seven genes with m.value < -1 (less than 2-fold decrease) all are consistent with those in RNA-seq data.

In the transition from K+6 to K+12, 111 genes were up-regulated DEGs in the microarray data. Of these genes, 80 genes (72.1%) showed similar changes in RNA-seq data, but 23 genes were down-regulated in RNA-seq data (type B in S5 Table). In the same transition, 59 genes were down-regulated in the microarray data, and 31 genes (52.5%) of them showed similar changes in RNA-seq data. Changes in expression of 22 genes (37.3%) were inconsistent with those in RNA-seq data (type C in S5 Table). DEGs in the microarray data were shown in MA plots, and also mapped in MA plots of TCC (S5 Fig). The up-regulated DEGs between K and K+6 in microarray data were mapped to high expression regions in MA plots of TCC. The down-regulated DEGs between K+6 and K+8 in microarray data were also mapped to high expression regions in MA plots of TCC. These suggest that highly expressed genes tend to be detected as DEGs in microarray experiments.

Different strains were used for sampling in these platforms: a conventional dikaryotic strain for microarray data [24] and strain #326 for RNA-seq data. In addition, tissues used in RNA-seq experiments contained additional tissues: tramal tissue and veil cells (S1 Fig). Inconsistency in gene expression between platforms might reflect the differences in strains and tissues used in both experiments. Therefore, it is possible that genes listed in S5 Table contain genes specifically expressed in dikaryons (types A and B in S5 Table) or tramal and veil cells (Type C in S5 Table). Thus, although most of the genes show consistent changes in both platforms, the presence of genes inconsistent with each is not surprising.

### 5. Changes in expression levels of genes previously reported

To further validate RNA-seq data, we examined RNA-seq data for genes whose expression levels were previously reported. The expression levels in RNA-seq data were given in RPKM values (S1 Dataset).

The three genes, *dst1*, *dst2* and *Cc*.*wc-2*, have been reported to be involved in photomorphogenesis of this fungus [7, 37–39]. These genes show similar expression patterns with each other (S6A Fig), consistent with the results in previous reports. High level of *dst2* expression in 3_sPri suggests a large amount of the Dst2 protein in this stage, which should make the primordia highly sensitive to blue light as the trigger for development into the fruiting body maturation stage.

The *eln3* gene encodes a predicted glycosyltransferase involved in stipe elongation [40]. There are three *eln3* paralogs in the genome [21], whose expressions are up-regulated in the stipe (S6B Fig), suggesting that these glycosyltransferases are involved in stipe elongation. Indeed, the *eln6* mutant which fails to elongate the stipe has been found to carry a mutation in CC1G_04713 (unpublished data).

The *ich1* gene was reported to be expressed in the primordia and the cap, and required for formation of the cap tissue [1]. In RNA-seq data, the *ich1* gene shows high expression in the primordia and the cap (S6C Fig), which is similar pattern to that previously reported. Although hyphal knots and primordial shafts form without *ich1* function [1], high expression level of *ich1* in hyphal knots suggest that *ich1* function would be required for cap differentiation in the early stage of wild-type fruiting. Expression of the *eln2* gene, which encodes a cytochrome P450 and whose mutation affects morphology of fruiting body primordia, showed expression patterns consistent with that previously reported [41]. The *clp1* gene has been found to be essential for clamp cell formation and expressed in strain #326 [18], consistent with RNA-seq data (S6D Fig).

Expression patterns of some genes appear to be inconsistent with the results previously reported. For example, *exp1* is required for cap expansion and has been reported to show relatively low expression in the vegetative mycelium. Our RNA-seq data indicate that *exp1* is expressed at moderate levels in the vegetative mycelium (S6D Fig). Similar expression levels of *exp1* have been reported in previous RNA-seq experiments using strain #326 [21]. It is possible that some genes in strain #326 exhibit expression patterns different from the wild-type dikaryon, because the mutated *A* mating type factor in this strain produces a fusion protein with the constitutive activity [15].

### 6. GO analysis of DEGs

Functional annotation clustering of DEGs was performed through the web-based interface of the DAVID Knowledgebase [36]. The number of annotation clusters and typical terms of the top three clusters with high enrichment score are listed in Table 2. Complete lists of enriched annotation terms in each transition are provided in S6 Table (also in S1 Dataset). When similar annotation terms are enriched in both up-regulated and down-regulated DEGs in a particular transition, amounts of proteins with similar functions might dynamically change in the transition. Such annotated terms are also shown in Table 2.

**Table 2:** GO analysis of DEGs in each transition.

Transition		DEG	Cluster	Enriched Top 3 annotation
1–2	Up	408	11	phospholipid biosynthetic process
				O-methyltransferase activity
				extracellular region **
	Down	134	3	Cytochrome P450
				Hydrophobin **
				adenyl nucleotide binding
2–3	Up	2	0	-
	Down	3	0	-
3–4	Up	85	1	adenyl nucleotide binding **
	Down	38	2	adenyl nucleotide binding **
				Hydrophobin
4–5	Up	148	3	structural constituent of ribosome
				zinc ion binding
				FAD binding
	Down	590	15	Hydrophobin, fungi
				phospholipid biosynthetic process
				septin complex
5–6	Up	949	38	DNA repair
				Chaperonin Cpn60/TCP-1
				glucose catabolic process **
	Down	1660	45	Hydrophobin
				Cytochrome P450
				polysaccharide metabolic process **
6–8	Up	2471	82	transport
				FAD binding
				Cytochrome P450
	Down	2034	64	RNA recognition motif, RNP-1
				RNA processing
				structural constituent of ribosome
8–10	Up	3000*/3465	93	transmembrane
				FAD binding
				Cytochrome P450
	Down	3000*/3197	112	structural constituent of ribosome
				WD40 repeat
				vesicle-mediated transport
10–12	Up	1743	62	nitrogen compound biosynthetic process
				cellular amino acid biosynthetic process
				Alcohol dehydrogenase GroES-like
	Down	1701	63	ATP binding
				FAD linked oxidase, N-terminal
				Galactose oxidase, beta-propeller
5–7	Up	3000*/3301	118	transport
				FAD binding
				ATP synthesis coupled proton transport
	Down	3000*/3569	83	structural constituent of ribosome
				RNA processing
				RNA recognition motif, RNP-1
7–9	Up	457	30	proteasome complex
				AMP-dependent synthetase and ligase
				Calcium-binding EF-hand
	Down	326	10	steroid metabolic process
				lipase activity
				lipid biosynthetic process
9–11	Up	2088	90	transport
				vitamin B6 binding
				Cytochrome P450
	Down	2356	91	structural constituent of ribosome
				WD40 repeat
				ribosome biogenesis
11–13	Up	136	5	Cytochrome P450 **
				Zinc finger, C2H2-type
				metal ion binding
	Down	192	7	Cytochrome P450 **
				FAD binding
				structural constituent of ribosome

* indicates that top 3000 genes were used for GO analysis.

** indicates that similar terms are annotated in both up- and down-DEGs.

**Fig 5:**
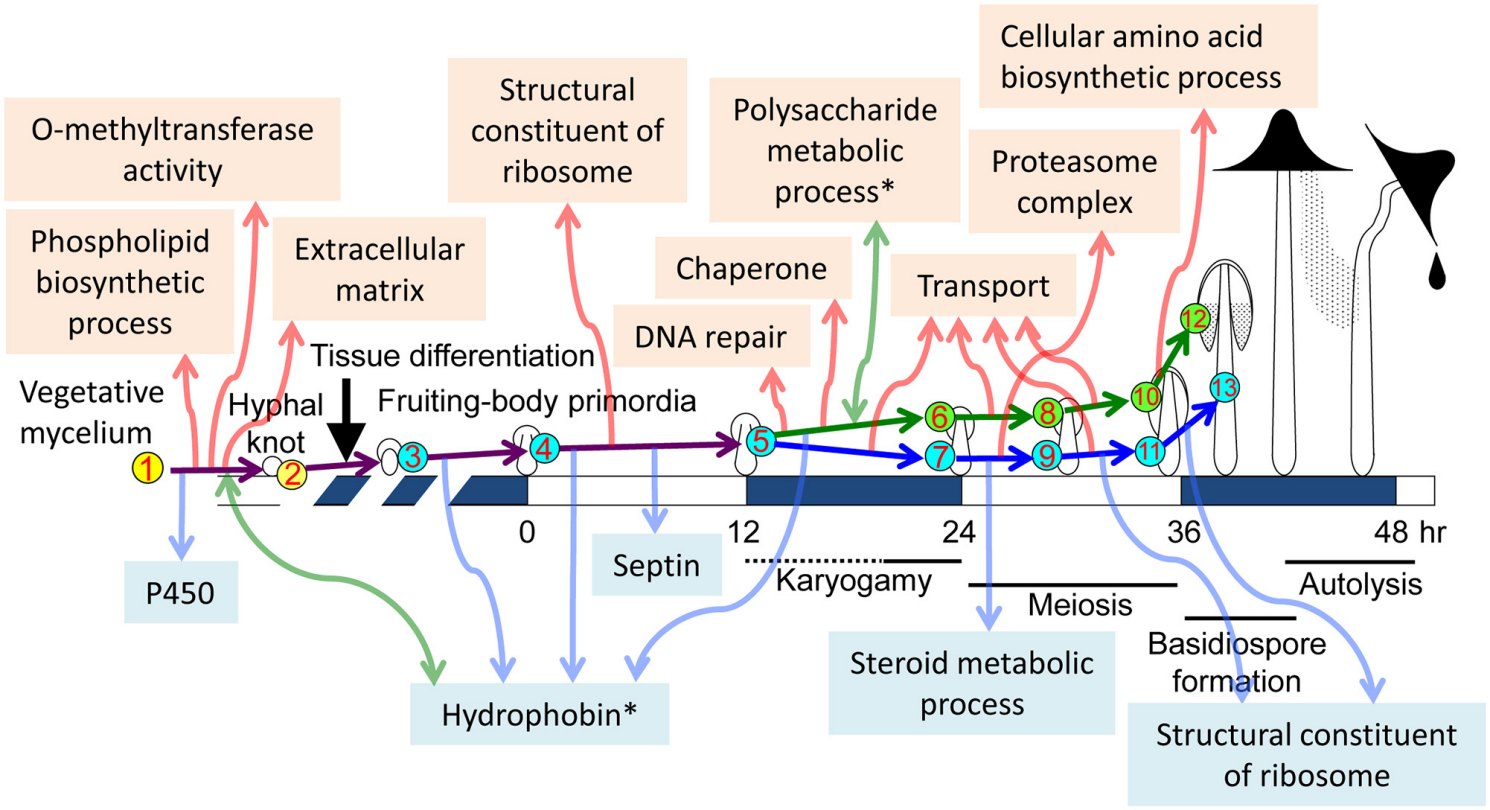
A model for significant developmental transitions based on GO analysis. Notable events among top 3 categories are depicted in fruiting. Events detected by up-regulated DEGs and down-regulated DEGs are indicated in the upper part with upward red arrows and in the lower part with downward blue arrows, respectively. The asterisks and bidirectional light green arrows indicate that similar annotation terms are enriched in both up-regulated and down-regulated DEGs.

A model for significant developmental transitions is presented on the basis of GO analysis (Fig 5). Cytochrome P450 enzymes, which might be involved in degradation of nutrients, are down-regulated in the transition from 1_Mycelium to 2_Knots. In hyphal knots, membrane components may differ from vegetative mycelium through phospholipid biosynthetic processes. Extracellular components in hyphal knots, including cell walls, hydrophobins and galectins [42], dynamically change, suggesting that these changes allow hyphal knot cells to communicate with each other to form proper multicellular pattern in the early stage of fruiting. A variety of hydrophobins have been reported to be developmentally expressed in fruiting of various mushrooms [43–47]. GO analysis indicates that expression of different hybrophobin genes are also developmentally regulated in *C*. *cinerea* (Table 2, S6 Table). Many O-methyltransferases, including Ich1 (S6C Fig), would have important roles in this transition, although the substrates are not yet known. In fruiting body primordia, the activity of many proteins might be regulated by FAD binding. Ribosome biogenesis is also pronounced between 4_0hrPri and 5_12hrPri. In contrast, expression of septin genes decreases between 4_0hrPri and 5_12hrPri. This decrease might imply temporary arrest of cell growth, which is released by proper light conditions and completes in the final maturation stage [13]. Genes necessary for premeiotic DNA replication and meiotic recombination are induced prior to karyogamy (nuclear fusion), and are included in the DNA repair category observed between 5_12hrPri and 6_24hrCap as reported previously [24]. Polysaccharide metabolic processes dynamically change in this transition, suggesting preparation for the dramatic enlargement of the cap (known to involve changes in glycogen metabolism [48]) in the next transition (6_24hrCap to 8_30hrCap). Chaperone and transport activities are up-regulated prior to enlargement of fruiting bodies, suggesting that many components are transported from vegetative mycelia to fruiting bodies and accumulated in cells to allow rapid cell expansion in the final stage of fruiting. In 12_39hrCap, cellular amino acid biosynthetic process is activated, consistent with previous reports [49, 50]. Lipid biosynthesis and steroid metabolic processes are down-regulated between 7_24hrStipe and 9_30hrStipe, prior to rapid stipe elongation. These down-regulations might change components of the plasma membrane and allow stipe cells to rapidly elongate in the following stage. The proteasome complex is activated in the stipe cells of this transition, suggesting that degradation of proteins occurs to yield energy for stipe elongation. This might correlate with down-regulation of ribosome biogenesis, saving energy. Thus, GO analysis of DEGs revealed many cellular events and processes in each transition on the basis of changes at the transcriptional level. Although we can discuss annotated terms enriched in DEGs, many genes without annotation are included among top 20 DEGs, as shown in S3 Table. Further experiments will be required to examine the function of DEGs without any annotation in each transition. In addition, most of genes and cellular events revealed by GO analysis might be located downstream of the cascades triggered by key signals and regulators, whose identification would be a future challenge.

### 7. Transcription factor candidates

**Fig 6:**
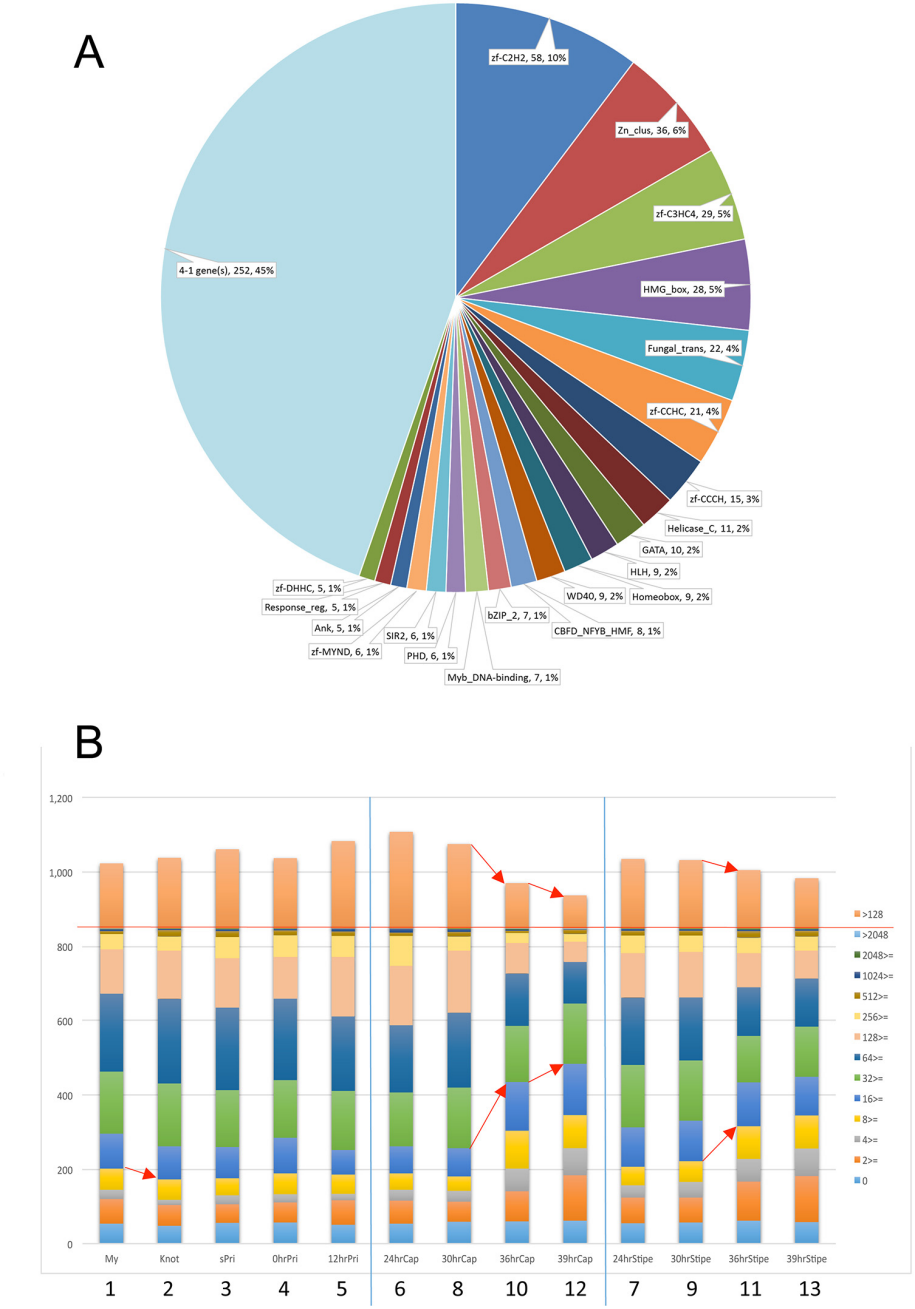
Transcription factor candidates in *C*. *cinerea* and changes in the expression levels. (A) Relative distribution of Pfam domains in the TFCs. A total of 848 TFCs were sorted on the basis of Pfam domains, and the number and percentage of each Pfam are indicated. (B) A histogram dividing 848 transcription factor candidates into bins based on their RPKM values. Red line shows a total number of the TFC genes, 848. Red arrows indicate notable changes.

Transcription factors are key regulators of gene expression. Even when expressed at low levels, they can influence expression of many target genes. Since RNA-seq can detect a wide dynamic range of transcription levels and differential expression patterns, we focused on transcription factors that regulate developmental processes. The kogdefline annotations were keyword-filtered and combined with Pfam annotations by the Broad Institute, yielding a list of 848 genes annotated as transcription factor candidates (TFCs). Pfam analysis of transcription factors has been performed on both ascomycete and basidiomycete genomes [51, 52]. The TFCs of *C*. *cinerea* were also classified on the basis of Pfam domains, resulting in 564 TCFs with Pfam domains (Fig 6A). Among the 564 TCFs, the C2H2 family (PF00096), Fungal Zn(2)-Cys(6) binuclear cluster domain (PF00172) family and C3HC4 family (PF00097) occupy 21% of TFC families in this fungus.

The TFCs were grouped based on ranges in RPKM, and change in frequency of each group was examined in developmental stages/tissues (Fig 6B). In the transition from 8_30hrCap to 10_36hrCap, the number of TCFs with RPKM>128 decreases and the number of TCFs with RPKM<16 increases. To see changes among the groups, RPKM values vs. m.value plots were depicted for each transition (S7 Fig). The number of TFC genes with high RPKM values decreases, and the number of genes with low RPKM values increases. It appears that changes in expression of TFC genes in the 8_30hrCap to 10_36hrCap transition leads to a large number of DEGs and the distinct expression patterns in the cap of the final stage (Fig 3).

**Fig 7:**
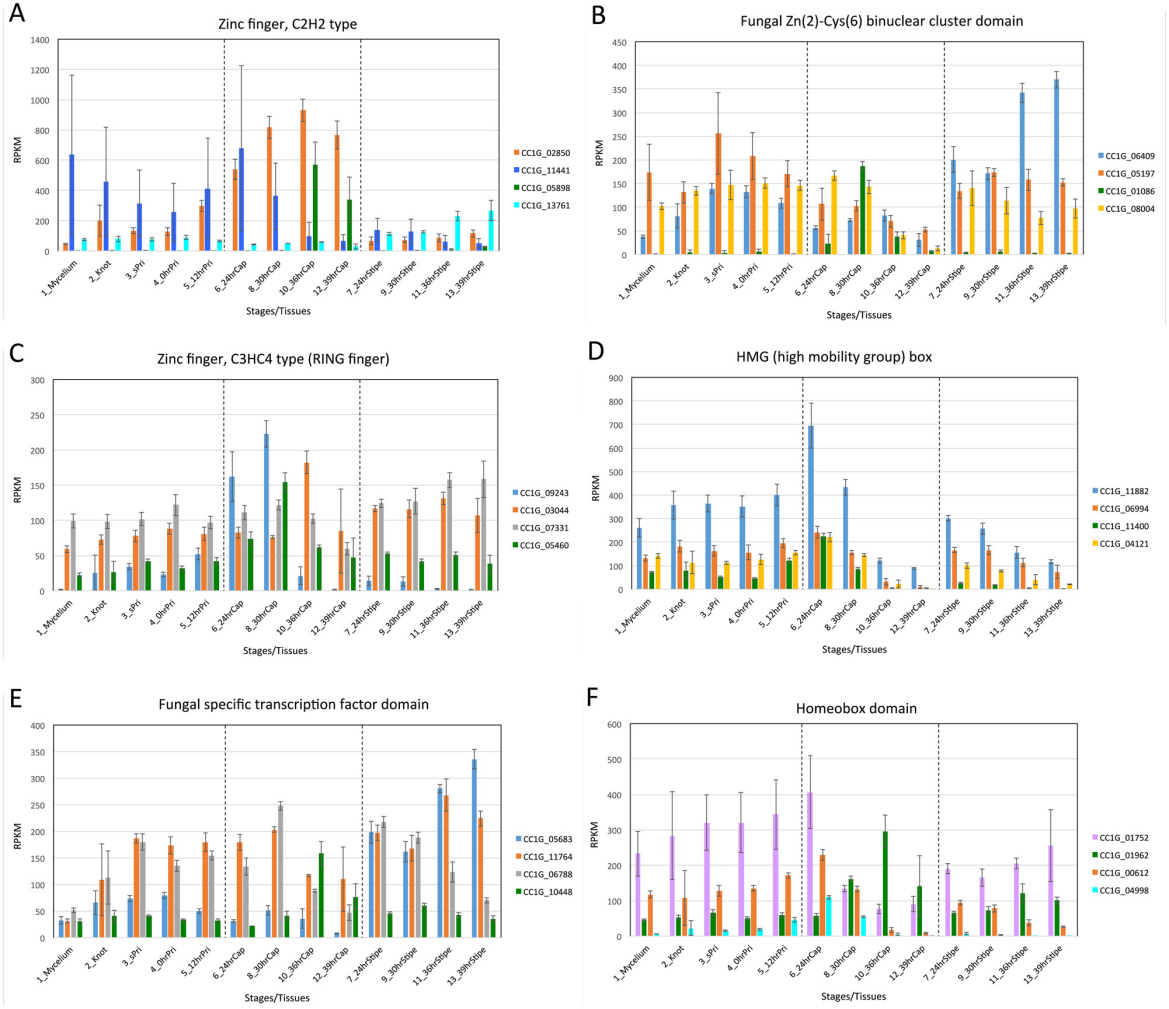
Changes in gene expression of TFCs with the same Pfam domain. (A) Top 4 genes of C2H2 transcription factors. (B) Top 4 genes of fungal Zn(2)-Cys(6) binuclear cluster domain. (C) Top 4 genes of zinc finger, C3HC4 type (RING finger). (D) Top 4 genes of HMG (high mobility group) box. (E) Top 4 genes of fungal specific transcription factor domain. (F) Top 4 genes of homeobox domain.

As shown in Fig 6B and S7 Fig, expression of TFCs changes during fruiting. Fig 7 shows changes in expression levels of top 4 genes of TFCs in each group classified on the basis of Pfam domains. To facilitate the detection of changes in expression levels, genes with similar expression levels were depicted in the same graph. These changes in expression of TFCs indicate that different types of TFCs are involved in each transition.

The Velvet regulons have been found to control sexual vs. asexual development in the ascomycete *Aspergillus nidulans* [53, 54], and expression of the orthologs in the basidiomycetes has also been examined [21]. Expression levels of genes encoding Velvet-associated proteins also show characteristic changes during fruiting (S8A–S8G Fig). The transcription factor genes, *fst3*, *fst4*, *bri1*, *hom1*, *hom2*, *c2h2* and *gat1*, have been investigated in fruiting body formation of *Schizophyllum commune* [55] and *C*. *cinerea* [21]. The transcription factor genes, *nit2/areA*, *nmr1* and *fox1* have been investigated in a plant-pathogenic basidiomycete *Ustilago maydis* [56, 57]. Expression levels of *C*. *cinerea* orthologs of these transcription factor genes were also examined (S8H and S8J Fig). The genes showing expression pattern with a peak might encode transcription factors that trigger particular cellular events, such as sporogenesis which might require many metabolic changes as predicted from Fig 3, or shown in Fig 5. However, it is also possible that the gene whose expression levels are relatively constant could receive environmental and internal signals and influence gene expression only after receiving such signals. A future challenge will be elucidation of precise transcriptional networks linking cellular events, in which ChIP-seq experiments using TFs as bait should be useful to accurately identify transcription factor binding sites (TFBS) in DEG promoters.

### 8. Potential role for antisense RNA in developmental regulation

**Fig 8:**
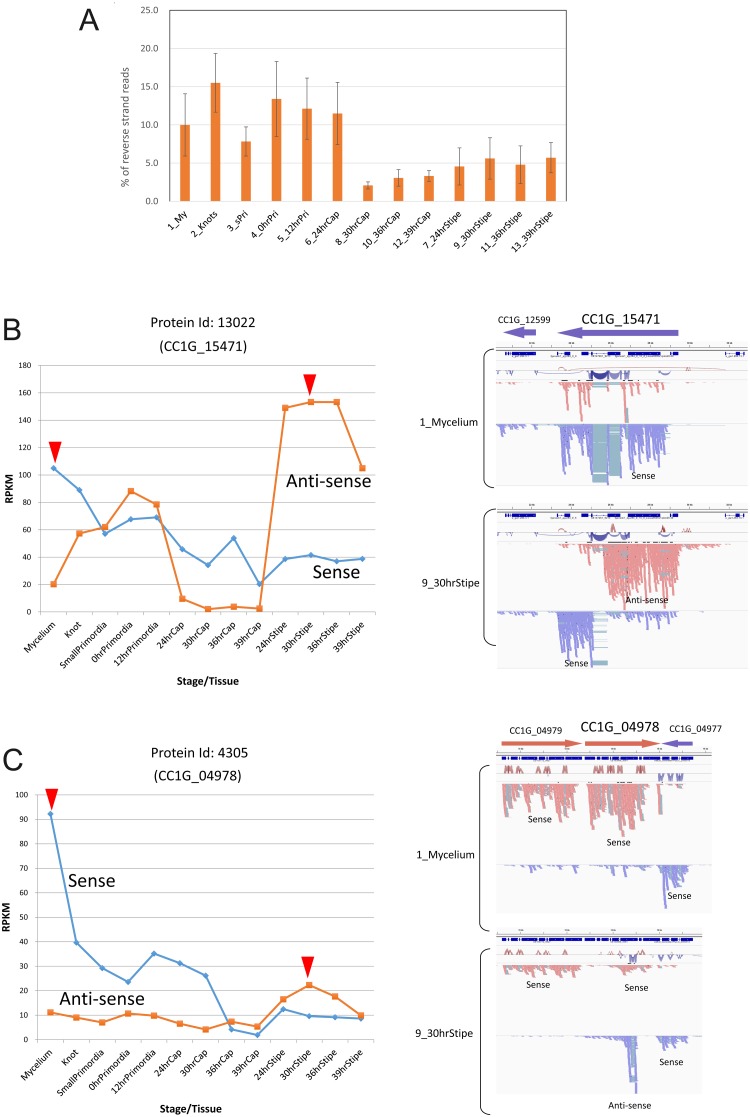
Antisense transcripts. (A) Change in the percentages of the antisense reads. (B) Antisense reads in Protein Id: 13022 (CC1G_15471). Left panel: Change in sense and antisense RPKM values. Red arrowheads indicate the points compared in the right panel. Right panel: R2 reads were mapped to the genomic sequences and separated based on read strands in IGV. (C) Antisense reads in Protein Id: 4305 (CC1G_04978), as shown in (B). Mapping of R1 reads showed similar patterns to those of R2 reads shown.

Natural antisense transcripts (NATs) have been described in most of eukaryotes [58–61]. RNA-seq libraries in this study were constructed by synthesizing second-strand cDNA using dUTP and degrading them so that sense and antisense transcripts could be distinguished. The percentages of reads mapped to the reverse strand of all gene models changed during fruiting (Fig 8A), suggesting that expression of antisense transcripts was developmentally regulated. Although the antisense counts are inevitably contaminated with the counts for the second strand of sense transcripts, we reasoned that if expression of the antisense transcript is regulated independently, the forward and reverse counts would not correlate. Accordingly, we calculated the correlation coefficient between the forward and reverse counts for each gene, and identified a total of 2,386 genes with negative correlation coefficients (S7 Table).

Using IGV, expression patterns of the genes with low correlation coefficients were observed to determine the location of the antisense transcripts within the gene. We were able to find obvious antisense transcripts in many genes, including Protein Id: 13022 (CC1G_15471) and Protein Id: 4305 (CC1G_04978) (Fig 8B and 8C). To confirm direction of the transcripts, we mapped reads to the genomic sequences and grouped them by direction (Fig 8B and 8C, right panels). The presence of introns in antisense transcripts also allows us to know the direction of the transcripts. CC1G_15471 encodes the predicted nuclear receptor coregulator SMRT/SMRTER, containing Myb-like domains, detected by kogdefline. The corresponding antisense transcript is expressed in the early stage of fruiting body formation, down-regulated in the cap, and up-regulated in the stipe (Fig 8B). The gene CC1G_04978 encodes a septin, Cc.AspE [13]. The antisense transcript in this gene becomes expressed before stipe elongation (Fig 8C). Other antisense transcripts were identified by negative correlation and the presence of introns in antisense transcripts (S9 Fig). The sense and antisense transcripts in S9 Fig are also developmentally regulated. In *Aspergillus nidulans*, inspection of transcriptome data revealed NATs, whose roles in transcriptional regulation have been investigated [62]. Experiments using strains defective in RNAi suggested that roles of NATs are independent of RNAi [62]. In *Saccharomyces cerevisiae* and *Aspergillus nidulans*, the majority of antisense transcripts have been reported to be expressed from the 3’ region of each annotated gene [59, 62]. Of 4 examples shown in Fig 8 and S9 Fig, two genes, CC1G_04978 and CC1G_01380, also express 3’ biased antisense transcripts, suggesting general roles for such NATs. Further studies will be required to examine roles of NATs in functional regulation of genes and fruiting body development of *C*. *cinerea*.

## Conclusions

The rapid, synchronous, light-regulated development in the model mushroom *Coprinopsis cinerea* can be dissected experimentally, allowing us to uncover the complex regulatory network that underlies fruiting body formation. Analyses of fruiting body development in this model mushroom using strand-specific RNA-seq revealed changes in expression of many gene models, providing clues for further transcriptomics, proteomics and metabolomics in basidiomycetes. GO analysis of DEGs in each transition revealed an overall developmental framework and many notable cellular events. We focused on transcription factor candidates (TFCs), which were identified using several methods, and sorted them on the basis of Pfam domains. We observed dramatic changes in gene expression of some of TFCs, also providing clues to identify specific transcriptional networks. We also observed several examples of stage-specific natural antisense transcripts (NATs), which are likely to provide important insights into development in less tractable basidiomycetes, and eukaryotes in general.

## Supporting Information

S1 Fig Developmental lineages among 13 stages/tissues. The number in a circle corresponds to the 13 stages/tissues shown in Fig 1. Each sample used for RNA-seq contains multiple tissues as shown. The light conditions to stimulate fruiting body development are shown in the upper region. Red arrows indicate flows of tissue differentiation. The dark period between 5_12hrPri and 6_24hrCap-7_24hrStipe is required to complete the maturation stage, and no dark period causes the abortive fruiting bodies [6, 7]. The asterisks indicate the lamella and gill tissues used for microarray analysis previously reported [24]. The microarray data derived from K, K+6, and K+12, were compared with RNA-seq data of 6_24hrCap, 8_30hrCap, and 10_36hrCap, respectively.
(TIF)

S2 Fig MM plots of historical and transitional differences. M.values in TCC normalization were plotted. The x-axis indicates m.values between 1_My and the previous stage (defined as the historical difference), and the y-axis indicates m.values between the indicated stage and the next stage (defined as the transitional difference). Red dots show genes with more than 4 m.values in the transitional difference, indicating DEG-type A. Purple dots show genes with more than 4 m.values in the historical difference and less than -4 m.values in the transitional difference, indicating DEG-type B. Green dots also show genes with less than -4 m.values in the transitional difference.
(TIF)

S3 Fig Validation of RNA-seq data. Log_2_ transformed ratio of gene expressions to that of β-tubulin. The x-axis and y-axis indicate the ratios in qPCR and RPKM of sense transcripts, respectively.
(TIF)

S4 Fig Comparison of expression profiles between microarray and RNA-seq data. Scatter plots were depicted using the averages of log transformed expression values at three time points, K, K+6, K+12, in microarray and RNA-seq data. Microarray data were normalized and transformed by vsn. RNA-seq data are given in log_2_ transformed RPKM values without RPKM = 0. The number of genes depicted in the graphs of K, K+6 and K+12 are 10,555, 10,609 and 10,560, respectively.
(TIF)

S5 Fig Microarray DEGs in MA plots of microarray and RNA-seq data. DEGs detected in microarray analysis are mapped in MA plots of microarray and RNA-seq data. The up-regulated DEGs are indicated by orange dots, and the down-regulated DEGs are indicated by green dots. MA plots of RNA-seq data were depicted by TCC. (A) MA plots of microarray data between 6_24hrCap (K) and 8_30hrCap (K+6). (B) MA plots of microarray data between 8_30hrCap (K+6) and 10_36hrCap (K+12). (C) MA plots of RNA-seq data between 6_24hrCap (K) and 8_30hrCap (K+6). (D) MA plots of RNA-seq data between 8_30hrCap (K+6) and 10_36hrCap (K+12).
(TIF)

S6 Fig Changes in expression levels of genes previously reported. (A) Upper panel shows changes in expression of *dst1*, *dst2* and *Cc*.*wc2*. Lower panel shows that of *Cc*.*wc2*. (B) Upper panel shows changes in expression of three *eln3* paralogs. Lower panel shows that of *eln6*. (C) Changes in expressions of *ich1* and *eln2*. (D) Changes in expressions of *clp1* and *exp1*.
(TIF)

S7 Fig RPKM vs M plot of TFCs. The x-axis represents log_2_ transformed RPKM values, and y-axis shows m.value to the next stage. Untransformed RPKM values, 0 to 4096, are also indicated as a scale in the upper region of the graph. Red circles indicate genes responsible for notable changes shown in Fig 6B.
(TIF)

S8 Fig Changes in expression of Velvet-associated proteins and reported transcription factors. (A-G) Genes encoding Velvet proteins containing those previously reported [53, 54]. (H, I) Genes encoding transcription factors previously reported [21]. CC1G_01962 (Hom2) is also shown in Fig 7F.
(TIF)

S9 Fig Changes in expression levels of antisense transcripts. (A) Protein Id: 380868 (CC1G_01380), prediciting to encode U1 snRNP-specific protein C, produces an antisense transcript, which is up-regulated in the vegetative mycelium. To clearly show change in expression levels of the antisense transcript in the graph, RPKM values of the antisense transcript are multiplied by four. (B) Protein Id: 457015 (CC1G_07663), predicting to encode guanine nucleotide exchange factor, produces an antisense transcript, which is up-regulated in 4_0hrPri. To clearly show change in expression levels of the antisense transcript in the graph, RPKM values of the antisense transcript are multiplied by two.
(TIF)

S1 Text Sequencing and annotation of the #326 (*Amut Bmut pab1-1*) genome. (DOCX)

S1 Table Strand-specific count data. (XLSX)

S2 Table List of primers used for qRT-PCR. (XLSX)

S3 Table List of top 20 DEGs with the most significant changes in expression at each of the 12 transitions. (XLSX)

S4 Table Summary of comparisons between microarray and RNA-seq. (XLSX)

S5 Table List of genes with inconsistencies between microarray and RNA-seq data. (XLSX)

S6 Table GO analysis of DEGs using DAVID. (XLSX)

S7 Table List of genes with negative Fw-Rv correlation coefficients. (XLSX)

S1 Dataset RPKM data of sense reads and GO analysis of DEGs. VBA macros in sheet “All_GO” enable any designated gene in the list to be found, and VBA macros in sheet “Auto_Graph” enable expression patterns of any designated gene to be displayed.
(XLSM)
