# Supplementary figures and images for: Strand-Specific RNA-Seq Analyses of Fruiting Body Development in Coprinopsis cinerea
Source: PLoS One. 2015 Oct 28;10(10):e0141586. doi: 10.1371/journal.pone.0141586 (PMC4624876; doi:10.1371/journal.pone.0141586)

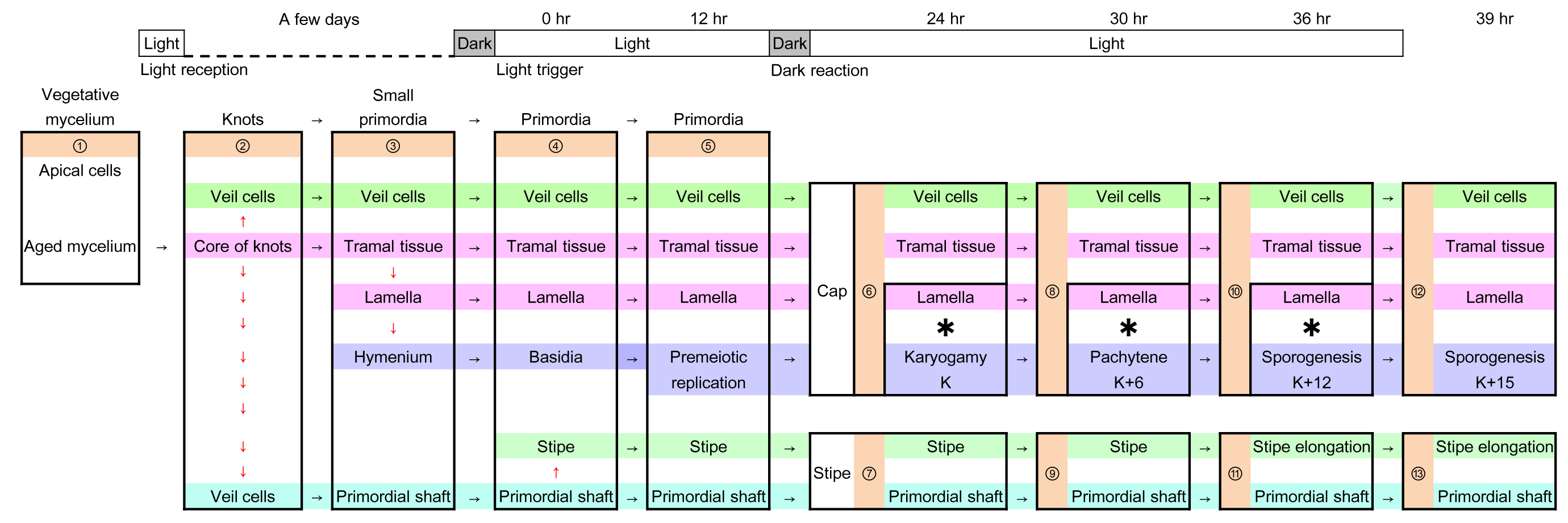

Supplement: S1 Fig — The number in a circle corresponds to the 13 stages/tissues shown in Fig 1. Each sample used for RNA-seq contains multiple tissues as shown. The light conditions to stimulate fruiting body development are shown in the upper region. Red arrows indicate flows of tissue differentiation. The dark period between 5_12hrPri and 6_24hrCap-7_24hrStipe is required to complete the maturation stage, and no dark period causes the abortive fruiting bodies [6, 7]. The asterisks indicate the lamella and gill tissues used for microarray analysis previously reported [24]. The microarray data derived from K, K+6, and K+12, were compared with RNA-seq data of 6_24hrCap, 8_30hrCap, and 10_36hrCap, respectively. (TIF) [file pone.0141586.s001.tif]

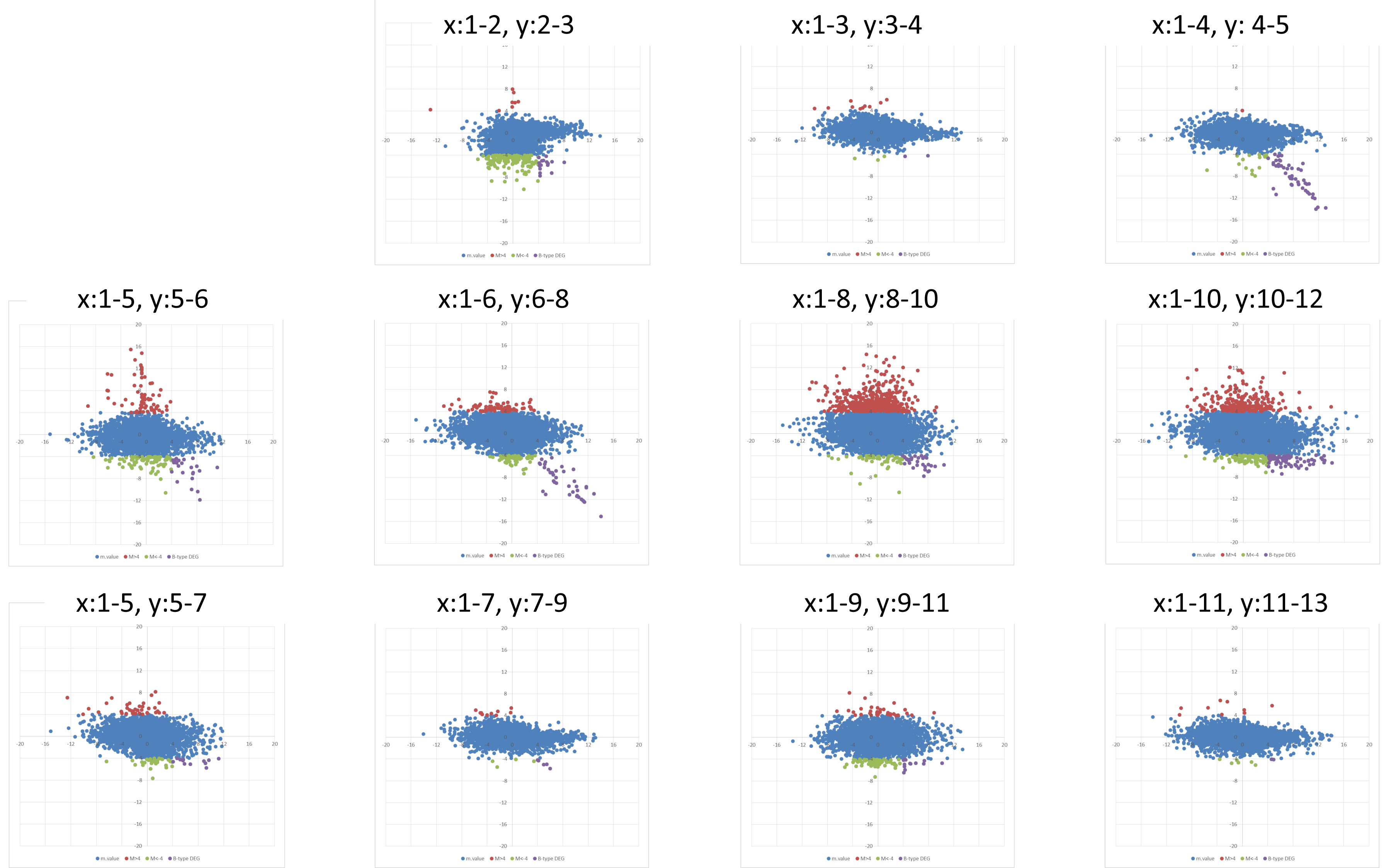

Supplement: S2 Fig — M.values in TCC normalization were plotted. The x-axis indicates m.values between 1_My and the previous stage (defined as the historical difference), and the y-axis indicates m.values between the indicated stage and the next stage (defined as the transitional difference). Red dots show genes with more than 4 m.values in the transitional difference, indicating DEG-type A. Purple dots show genes with more than 4 m.values in the historical difference and less than -4 m.values in the transitional difference, indicating DEG-type B. Green dots also show genes with less than -4 m.values in the transitional difference. (TIF) [file pone.0141586.s002.tif]

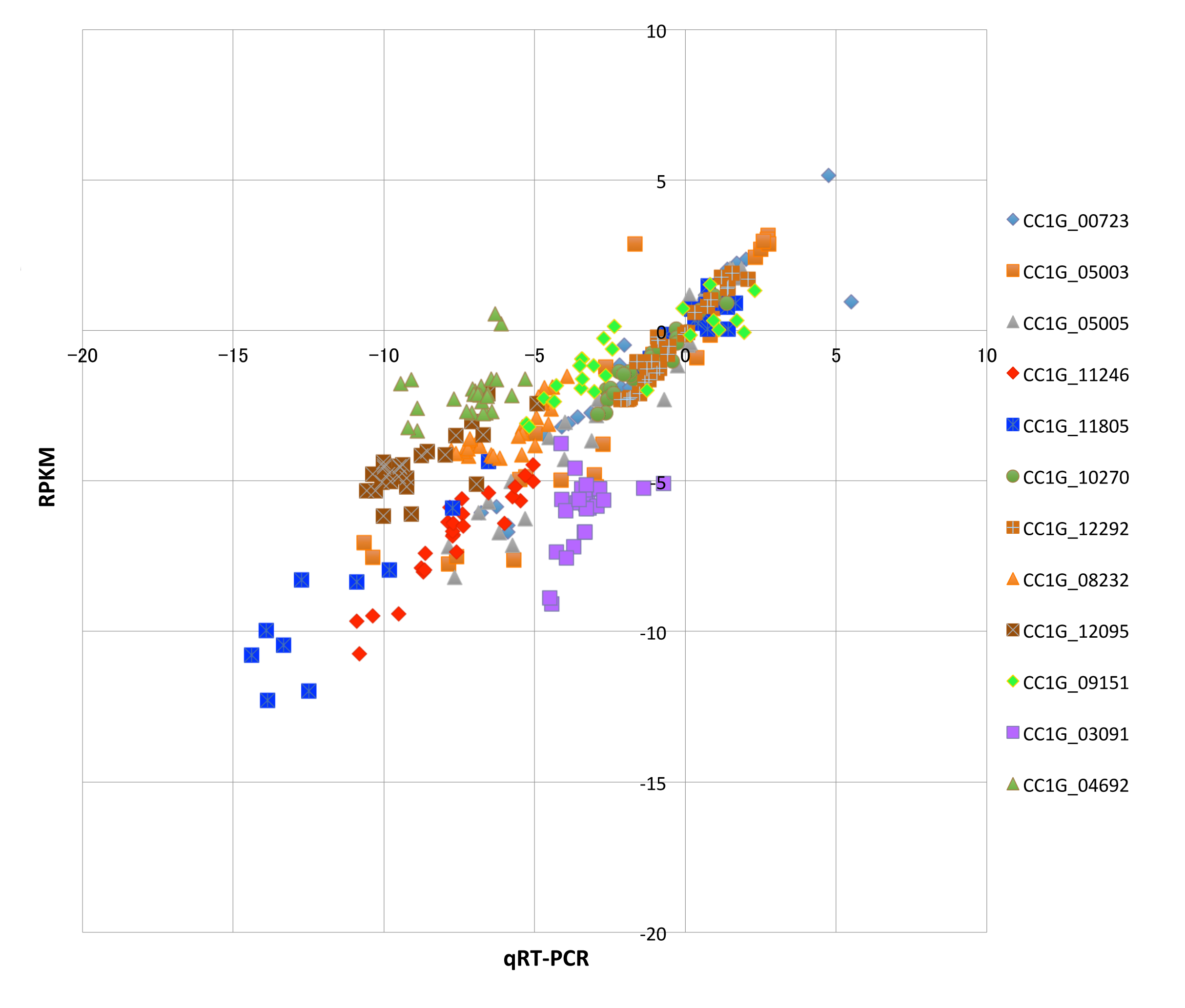

Supplement: S3 Fig — Log2 transformed ratio of gene expressions to that of β-tubulin. The x-axis and y-axis indicate the ratios in qPCR and RPKM of sense transcripts, respectively. (TIF) [file pone.0141586.s003.tif]

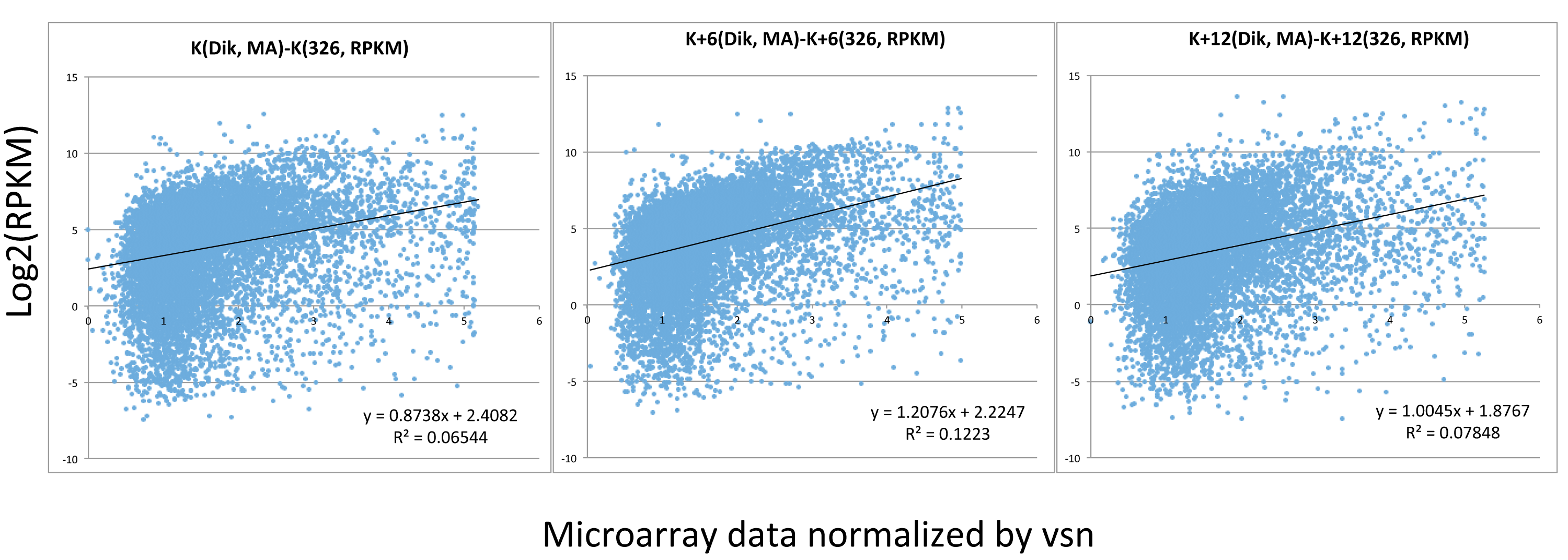

Supplement: S4 Fig — Scatter plots were depicted using the averages of log transformed expression values at three time points, K, K+6, K+12, in microarray and RNA-seq data. Microarray data were normalized and transformed by vsn. RNA-seq data are given in log2 transformed RPKM values without RPKM = 0. The number of genes depicted in the graphs of K, K+6 and K+12 are 10,555, 10,609 and 10,560, respectively. (TIF) [file pone.0141586.s004.tif]

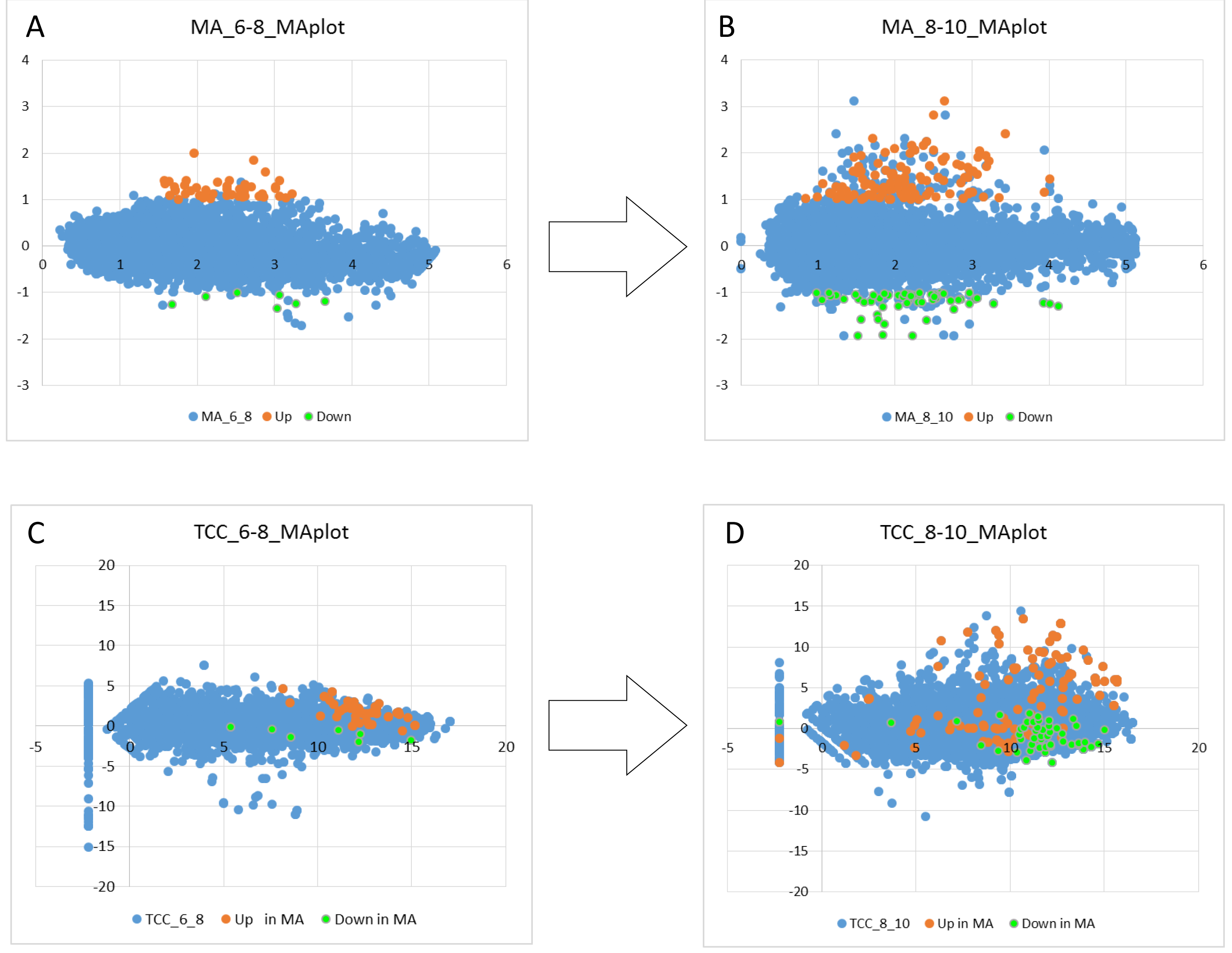

Supplement: S5 Fig — DEGs detected in microarray analysis are mapped in MA plots of microarray and RNA-seq data. The up-regulated DEGs are indicated by orange dots, and the down-regulated DEGs are indicated by green dots. MA plots of RNA-seq data were depicted by TCC. (A) MA plots of microarray data between 6_24hrCap (K) and 8_30hrCap (K+6). (B) MA plots of microarray data between 8_30hrCap (K+6) and 10_36hrCap (K+12). (C) MA plots of RNA-seq data between 6_24hrCap (K) and 8_30hrCap (K+6). (D) MA plots of RNA-seq data between 8_30hrCap (K+6) and 10_36hrCap (K+12). (TIF) [file pone.0141586.s005.tif]

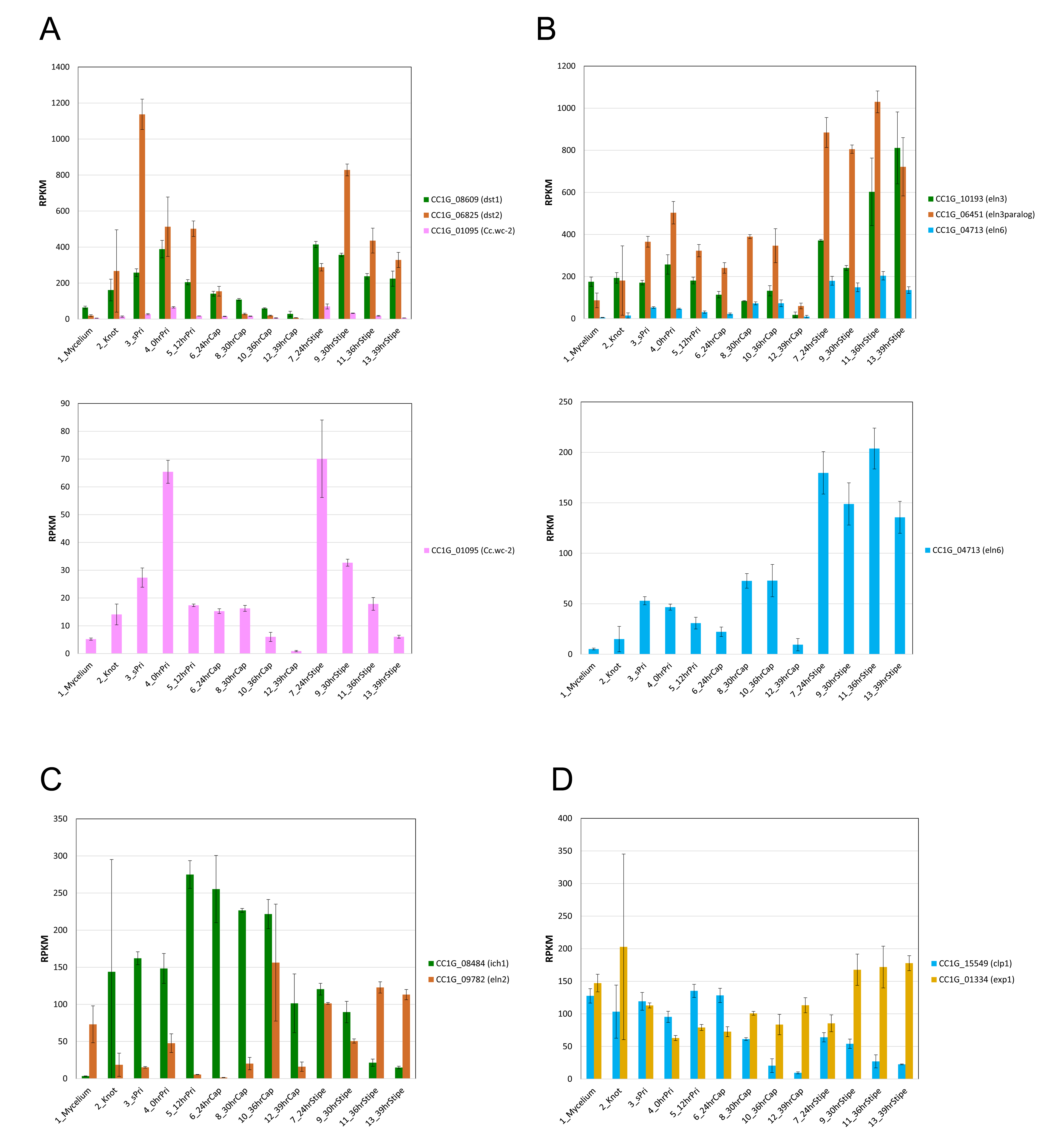

Supplement: S6 Fig — (A) Upper panel shows changes in expression of dst1, dst2 and Cc.wc2. Lower panel shows that of Cc.wc2. (B) Upper panel shows changes in expression of three eln3 paralogs. Lower panel shows that of eln6. (C) Changes in expressions of ich1 and eln2. (D) Changes in expressions of clp1 and exp1. (TIF) [file pone.0141586.s006.tif]

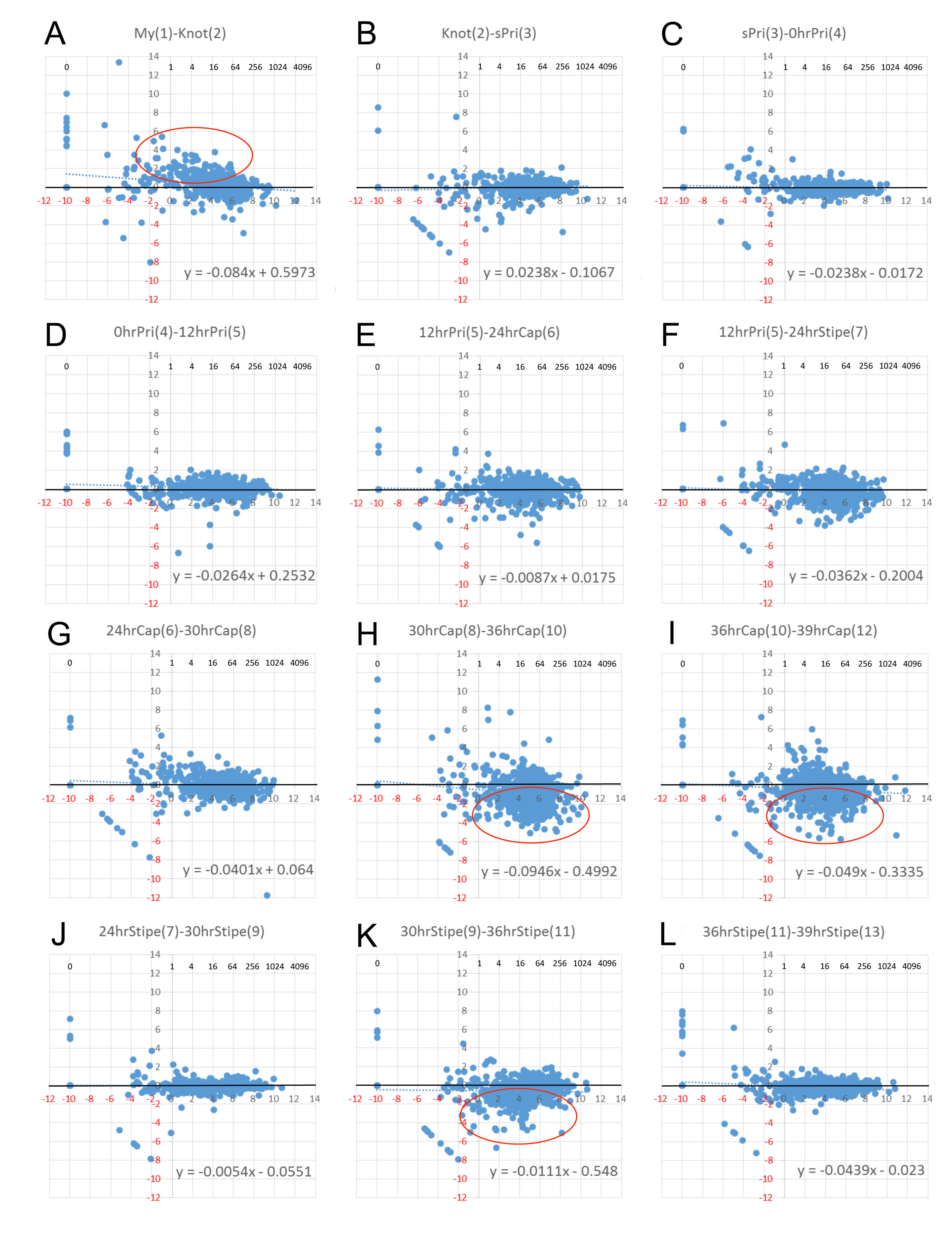

Supplement: S7 Fig — The x-axis represents log2 transformed RPKM values, and y-axis shows m.value to the next stage. Untransformed RPKM values, 0 to 4096, are also indicated as a scale in the upper region of the graph. Red circles indicate genes responsible for notable changes shown in Fig 6B. (TIF) [file pone.0141586.s007.tif]

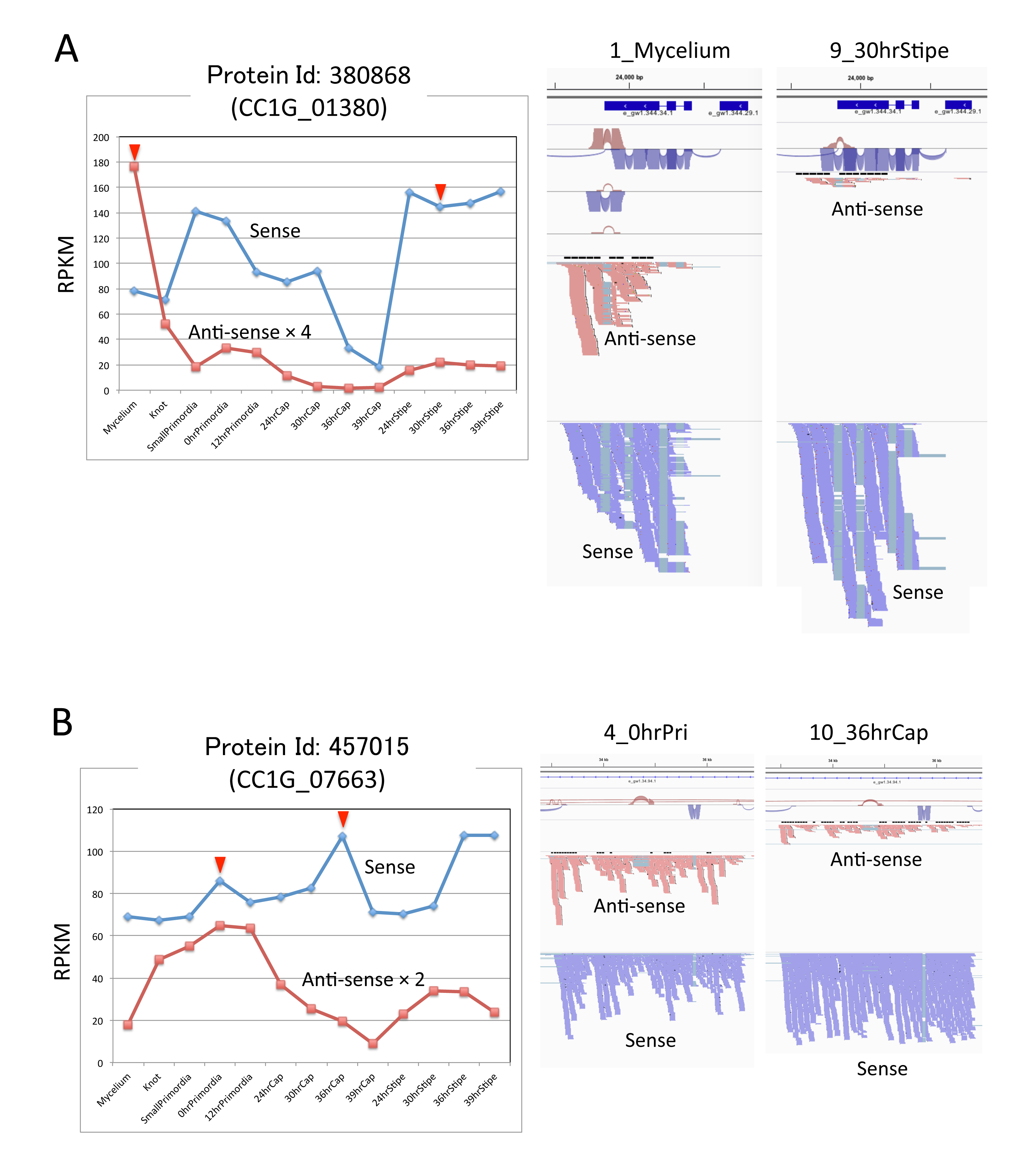

Supplement: S9 Fig — (A) Protein Id: 380868 (CC1G_01380), prediciting to encode U1 snRNP-specific protein C, produces an antisense transcript, which is up-regulated in the vegetative mycelium. To clearly show change in expression levels of the antisense transcript in the graph, RPKM values of the antisense transcript are multiplied by four. (B) Protein Id: 457015 (CC1G_07663), predicting to encode guanine nucleotide exchange factor, produces an antisense transcript, which is up-regulated in 4_0hrPri. To clearly show change in expression levels of the antisense transcript in the graph, RPKM values of the antisense transcript are multiplied by two. (TIF) [file pone.0141586.s009.tif]
