# Supplementary material for: Strand-Specific RNA-Seq Analyses of Fruiting Body Development in Coprinopsis cinerea
Source: PLoS One. 2015 Oct 28;10(10):e0141586. doi: 10.1371/journal.pone.0141586 (PMC4624876; doi:10.1371/journal.pone.0141586)
Supplement: S1 Text — (DOCX) [file pone.0141586.s010.docx]

**S1 Text.** Sequencing and annotation of the strain #326 (*Amut Bmut pab1-1*) genome

200ng of *Coprinopsis cinerea* *AmutBmut pab1-1* DNA was sheared to 270bp using the covaris E210 (Covaris) and size selected using SPRI beads (Beckman Coulter). The fragments were treated with end-repair, A- tailing, and ligation of Illumina compatible adapters (IDT, Inc) using the KAPA-Illumina library creation kit (KAPA biosystems). qPCR was used to determine the concentration of the library and were sequenced on the Illumina Hiseq. 2X150bp paired end reads from the single library were filtered for artifact and process contamination and assembled with Velvet (Zerbino and Birney, 2008). The resulting assembly was used to create a simulated long mate-pair library with 3kb insert, which was then assembled together with the original Illumina library using AllPathsLG release version R42328 (Gnerre et al., 2011). The final 34.1Mb assembly in 944 scaffolds and 1053 contigs was annotated using the JGI annotation pipeline as described previously (Kuo et. al., 2014). Briefly, the pipeline generates gene models at each locus using a variety of RNASeq-based, protein-based and *ab initio* gene predictors and selects a 'best prediction' at each locus based on gene expression, similarity to known proteins and domain content. The pipeline also annotates the gene models by predicting functional domains and exports the assembly and annotation to an interactive Portal in MycoCosm (Grigoriev et al. 2014). The *C.cinerea* *AmutBmut pab1-1* (#326) genome portal can be found at <http://genome.jgi.doe.gov/Copci_AmutBmut1>.

**References**

- Zerbino DR, Birney E. (2008) Velvet: algorithms for de novo short read assembly using de Bruijn graphs. Genome Res. 18(5):821-9.
- Gnerre S, Maccallum I, Przybylski D, Ribeiro FJ, Burton JN, Walker BJ, Sharpe T, Hall G, Shea TP, Sykes S, Berlin AM, Aird D, Costello M, Daza R, Williams L, Nicol R, Gnirke A, Nusbaum C, Lander ES, Jaffe DB. (2011) High-quality draft assemblies of mammalian genomes from massively parallel sequence data. Proc Natl Acad Sci U S A. 25;108(4).
- Kuo A, Bushnell B, Grigoriev IV (2014) Fungal genomics: sequencing and annotation. In: Fungi. Advances in Botanical Research, Vol. 70 (ed. Martin F), pp. 1–52. Elsevier, London.
- Grigoriev IV, Nikitin R, Haridas S, Kuo A, Ohm R, Otillar R, Riley R, Salamov A, Zhao X, Korzeniewski F, Smirnova T, Nordberg H, Dubchak I, Shabalov I. (2014) MycoCosm portal: gearing up for 1000 fungal genomes. Nucleic Acids Res. 42(1):D699-704.
